# Novel obesity and metabolic indices better predict non-alcoholic fatty liver disease in elderly T2DM patients: evidence from cross-sectional and longitudinal analysis

**DOI:** 10.3389/fmed.2025.1649466

**Published:** 2025-09-22

**Authors:** Mingjie Xu, Yushuang Wei, Lingyu Ye, Boteng Yan, Shengzhu Huang, Zengnan Mo, Mingli Li

**Affiliations:** ^1^Department of Urology, Hunan University of Medicine General Hospital, Huaihua, China; ^2^Center for Genomic and Personalized Medicine, Guangxi Key Laboratory for Genomic and Personalized Medicine, Guangxi Collaborative Innovation Center for Genomic and Personalized Medicine, Guangxi Medical University, Nanning, Guangxi, China; ^3^School of Public Health, Guangxi Medical University, Nanning, Guangxi, China; ^4^Institute of Urology and Nephrology, First Affiliated Hospital of Guangxi Medical University, Guangxi Medical University, Nanning, Guangxi, China

**Keywords:** NAFLD, MetS score, LAP, elderly, MetS (metabolic syndrome)

## Abstract

**Objective:**

The associations between obesity- and metabolism-related indices and the risk of non-alcoholic fatty liver disease (NAFLD) in elderly patients with type 2 diabetes mellitus (T2DM) remain unclear. This study aimed to investigate these associations and assess their predictive value for NAFLD in this high-risk population.

**Methods:**

A total of 789 elderly T2DM patients recruited between 2020 and 2022 were included in the cross-sectional analysis, and 382 patients without NAFLD were followed in the longitudinal cohort for a median of 25.37 months. Binary logistic regression and Cox models were used to assess associations between obesity- and metabolism-related indices and NAFLD risk. Kaplan–Meier curves, restricted cubic spline (RCS) models, subgroup analysis, and receiver operating characteristic (ROC) curves were applied to explore these relationships further.

**Results:**

In the cross-sectional analysis, all obesity- and metabolic-related indices were significantly and positively associated with NAFLD risk, with odds ratio (OR) ranging from 1.014 (95% CI: 1.010–1.018) for LAP to 3.288 (95% CI: 2.414–4.533) for WHtR. RCS analysis revealed significant nonlinear associations for LAP, MetS scores, VAI, CMI, METS-IR, and ABSI. In the cohort analysis, 67 participants developed NAFLD, with an incidence rate of 8.35 per 100 person-years. Baseline LAP (HR = 3.10, 95% CI: 1.48–6.51), and MetS scores (HR = 4.26, 95% CI: 1.99–9.11) were independently associated with increased risk of incident NAFLD. Subgroup analysis demonstrated consistent positive associations across most subgroups. Time-dependent ROC analysis showed that LAP had the highest AUCs at 24 months (AUC = 0.725).

**Conclusion:**

The findings from cross-sectional and cohort studies collectively supported that MetS score and LAP may be the most effective predictive indicators for the risk of NAFLD among Chinese elderly T2DM Patients.

## Introduction

Non-alcoholic fatty liver disease (NAFLD) is characterized by the accumulation of fat within hepatocytes, in the absence of secondary causes such as alcohol abuse, viral hepatitis, or inherited metabolic disorders (1). As a chronic and progressive liver condition, NAFLD can advance to hepatic decompensation and malignant outcomes such as hepatocellular carcinoma, imposing a substantial global disease burden (2, 3). In recent years, both the prevalence and incidence of NAFLD have continued to rise worldwide, with a pooled global prevalence estimated at 32.4% (4). Elderly individuals have garnered increasing attention as a high-risk population, given their greater susceptibility to metabolic disturbances (5). NAFLD commonly coexists with metabolic syndrome (MS) and type 2 diabetes mellitus (T2DM), forming a pathophysiological triad that synergistically accelerates the progression of atherosclerotic cardiovascular disease (6–8). Although NAFLD is highly prevalent worldwide, the underlying mechanisms driving its initiation and progression have not been fully characterized. Currently, there are no approved pharmacological treatments for NAFLD (9). However, early-stage NAFLD is potentially reversible, highlighting the critical importance of prevention (10). Considering the escalating public health impact of NAFLD and the demographic trend of an aging population in China, the identification of robust predictors for NAFLD in the elderly is essential for guiding preventive strategies and optimizing clinical management.

Obesity and metabolic dysfunction are widely recognized as key drivers in the development and progression of NAFLD (11). In the early stages of the disease, hepatic fat accumulation and functional impairment may be induced through alterations in lipid metabolism, insulin resistance, and abnormal fat distribution (12, 13). To more accurately evaluate obesity-associated metabolic dysfunction, a series of refined anthropometric and metabolic indices have been developed to address the limitations of conventional measures such as body mass index (BMI) and waist-to-height ratio (WHtR) in capturing metabolic risk. These indices include the metabolic score for insulin resistance (METS-IR), lipid accumulation product (LAP), visceral adiposity index (VAI), a body shape index (ABSI), body roundness index (BRI), cardiometabolic index (CMI), and the metabolic syndrome score (MetS score). In contrast to conventional indicators, these novel indices are constructed by integrating parameters related to body composition, lipid metabolism, and insulin sensitivity, and are considered to provide a more comprehensive assessment of cardiometabolic risk (14–17). Previous studies have demonstrated the predictive utility of these indices in metabolic disorders such as metabolic syndrome and T2DM (18–20). However, their performance in predicting NAFLD among elderly patients with T2DM has not been fully elucidated. It is considered necessary to evaluate these indices for NAFLD screening due to the elevated metabolic burden and altered fat distribution in this population.

Therefore, a cross-sectional analysis was conducted to examine the associations between these nine indices and NAFLD, followed by a prospective cohort analysis to further substantiate the observed relationships. This study may facilitate the identification of the most effective indicator for NAFLD risk prediction in elderly patients with T2DM and provide a scientific basis for stratified risk assessment and early intervention.

## Method

### Participants and study design

The data utilized in this study were extracted from the Basic Public Health Service (BPHS) management system, which provides primary healthcare services, including chronic disease management, health education, and routine physical examinations, to the local population. Individuals aged 65 years and older are entitled to free health services, including regular health check-ups and follow-up management. Participants under management have consented to the potential use of their health record data for scientific research purposes.

**Figure 1:**
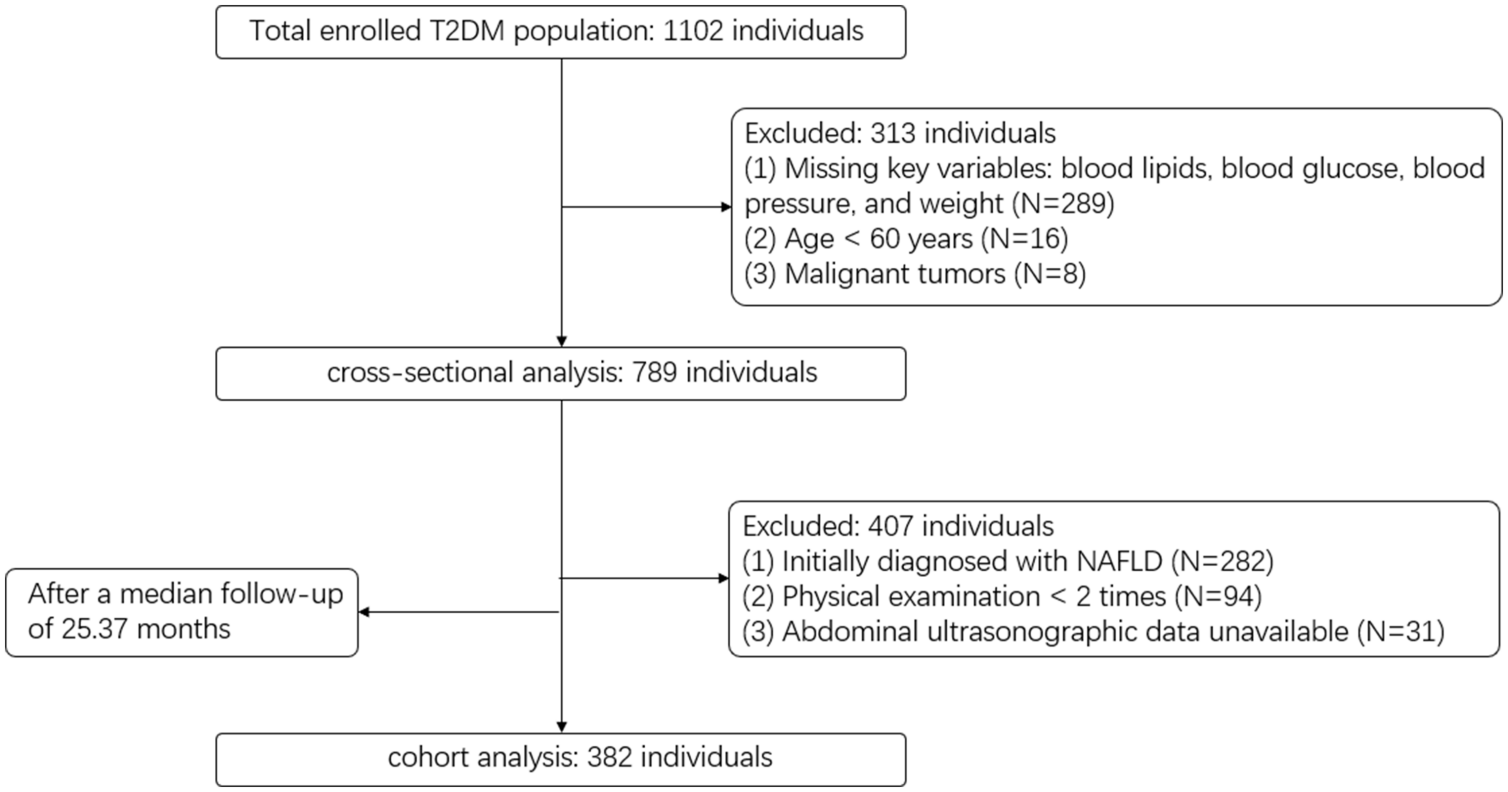
Flowchart of participant selection process for cross-sectional and cohort analyses.

We extracted electronic health records from the Wuliqiao Community Health Service Center, affiliated with the First People’s Hospital of Yulin City, Guangxi, covering the period from 2020 to 2024. Inclusion and exclusion criteria are detailed in Figure 1. In total, 789 participants were included in the cross-sectional analysis, and 382 were followed up and included in the cohort analysis.

Sample size calculations were performed using BMI as the representative variable in both the cross-sectional and cohort analyses, as it is the most widely used, easily obtainable, and well-established measure of obesity, and typically exhibits the smallest effect size among the nine indices, providing a conservative estimate. For the cross-sectional analysis, we reviewed the literature and found that the reported OR for BMI and NAFLD typically ranges from 1.2 to 1.4. Accordingly, we selected an OR of 1.3 as the assumed effect size. Using OR = 1.3, baseline probability (P0) = 0.3, R^2^ = 0.12, *α* = 0.05, power = 0.85, the required minimum sample size was estimated to be approximately 705. For the cohort analysis, we similarly used PASS software based on a Cox regression model, assuming a standard deviation of the exposure variable of 2.89, event rate = 0.17, regression coefficient = 0.1820, R^2^ = 0.12, *α* = 0.05, and power = 0.85. The required minimum sample size was initially estimated to be 217 without accounting for attrition. After adjusting for an anticipated 20% dropout rate, the required minimum sample size was approximately 272. The sample size in this study was sufficient to meet the minimum requirement.

### Calculation of obesity- and metabolism-related indices

Height and weight were measured using an Omron device with participants wearing light clothing and no shoes. Body weight was recorded to the nearest 0.1 kg, and height to the nearest 0.1 cm. Waist circumference (WC) was measured at the midpoint between the lower margin of the last palpable rib and the iliac crest. Hip circumference was measured using a flexible tape placed around the widest portion of the buttocks. Blood pressure was measured after the participant had rested in a seated position for at least 10 min, and the average of two consecutive measurements was recorded. The following nine anthropometric and metabolic indices were calculated based on baseline clinical and biochemical data:


$$\mathrm{BMI}=w\text{eight}/\text{heigh}t^{2}$$

WHtR=WC/height

$$\text{METS}-\mathrm{IR}=\ln[2\times \mathrm{FPG}+\mathrm{TG}]\times \frac{\mathrm{BMI}}{\ln(\mathrm{HDL}-C)}$$

$$\mathrm{LA}P_{\text{male}}=(\mathrm{WC}-65)\times \mathrm{TG}$$

$$\mathrm{LA}P_{\text{female}}=(\mathrm{WC}-58)\times \mathrm{TG}$$

$$\mathrm{VA}I_{\text{male}}=(\frac{\mathrm{WC}}{39.68+1.88\times \mathrm{BMI}})\times (\frac{\mathrm{TG}}{1.03})\times (\frac{1.31}{\mathrm{HDL}-C})$$

$$\mathrm{VA}I_{\text{female}}=(\frac{\mathrm{WC}}{36.58+1.89\times \mathrm{BMI}})\times (\frac{\mathrm{TG}}{0.81})\times (\frac{1.52}{\mathrm{HDL}-C})$$

$$\text{ABSI}=\frac{\mathrm{WC}}{\mathrm{BM}I^{2/3}\times \text{Heigh}t^{1/2}}$$

$$\mathrm{BRI}=364.2-365.5\times \sqrt{1-{\left(\frac{\mathrm{WC}}{2\pi }\right)}^{2}/{\text{Height}}^{2}}$$

$$\mathrm{CMI}=(\frac{\mathrm{TG}}{\mathrm{HDL}-C})\times \text{WHtR}$$

$$\begin{array}{l} \text{MetS}\_\text{score}=-3.1436+0.0258\times \mathrm{WC}+0.361\times \mathrm{TG}-0.9348\times \\ \mathrm{HDL}-C+0.0128\times \mathrm{MAP}+0.1224\times \mathrm{FBG} \end{array}$$

Body weight was measured in kilograms (kg), and height in meters (m). Waist circumference (WC) was recorded in centimeters (cm). FPG refers to fasting plasma glucose (mg/dL), TG to triglycerides (mg/dL), and HDL-C to high-density lipoprotein cholesterol (mg/dL). MAP represents mean arterial pressure, and FBG denotes fasting blood glucose, measured in millimoles per liter (mmol/L).

MS was determined using the Chinese Diabetes Branch of the Chinese Medical Association (CDS2013) criteria. MetS was defined as the presence of no less than three risk factors as follows: (1) abdominal obesity: WC ≥ 90 cm (men), WC ≥ 85 cm (female), (2) Elevated BP: BP ≥ 130/85 mmHg and/or those who have been diagnosed and treated for hypertension, (3) Elevated fasting glucose: FBG ≥ 6.1 mmol/L or 2hPG ≥ 7.8 mmol/L and/or have been Diagnosis of diabetes and treatment, (4) Elevated triglycerides: TG ≥ 1.7 mmol/L, (5) Reduced HDL-C: HDL-C < 1.04 mmol/L.

### Measurement of covariate variables

Trained medical personnel from the community health service center conducted standardized face-to-face interviews using structured questionnaires and collected fasting blood samples. The questionnaire obtained detailed information on participants’ sociodemographic characteristics, lifestyle behaviors, medical history, and medication use. Collected variables included gender, age, physical activity status, smoking status, and alcohol consumption. Medical history covered physician-diagnosed chronic diseases such as hypertension, diabetes, dyslipidemia, and cardiovascular disease. Information on current use of medications for chronic conditions, including antihypertensive, hypoglycemic, and lipid-lowering agents, was also collected. Fasting blood samples were used to assess basic biochemical parameters, including fasting blood glucose, lipid profile (total cholesterol, triglycerides, HDL-C, LDL-C), and renal function indicators such as serum creatinine.

### Definition of outcome measures

The diagnosis of NAFLD is based on two primary criteria (21): (1) exclusion of significant alcohol consumption, defined as a daily ethanol intake of less than 30 g for men and less than 20 g for women, along with the exclusion of other known causes of hepatic steatosis; and (2) imaging evidence of diffuse hepatic steatosis. Ultrasonographic features characteristic of NAFLD include: (a) increased hepatic echogenicity in the near field compared with the renal cortex (i.e., “bright liver”); (b) attenuation of far-field hepatic echoes; and (c) poor visualization of intrahepatic vascular and ductal structures (22). The diagnosis is made by integrating these imaging findings with the patient’s clinical history and exclusion criteria.

### Statistical analysis

Continuous variables were summarized as mean ± standard deviation (SD) for normally distributed data, or as median with interquartile range (IQR) for non-normally distributed data. Between-group comparisons were performed using Student’s t-test for normally distributed variables and the Mann–Whitney U test for skewed distributions. Categorical variables were presented as frequencies and percentages, with comparisons conducted using the chi-square test.

To facilitate model estimation and improve numerical stability, ABSI and WHtR values were multiplied by 10 prior to analysis due to their relatively small original magnitudes. In the cross-sectional analysis, binary logistic regression models were used to assess the associations between obesity and metabolic indicators with the presence of NAFLD. In the cohort analysis, Cox proportional hazards models were employed to evaluate the longitudinal associations between baseline exposures and incident NAFLD. We constructed a directed acyclic graph (DAG) with DAGitty.net to formally encode the hypothesized causal relationships among study variables, enabling systematic identification and visualization of confounders, mediators, and colliders informed by prior knowledge (Supplementary Figure S1). Both univariate and multivariable models were fitted, and the results were reported as odds ratio (OR) or hazard ratio (HR) with 95% confidence intervals (CIs). Kaplan–Meier survival analysis was performed to estimate the cumulative incidence of NAFLD across quartiles of exposure variables, and differences between groups were tested using the log-rank test. Restricted cubic spline (RCS) models were applied to explore potential nonlinear associations between continuous predictors and NAFLD risk, with subgroup analysis conducted based on key demographic and clinical characteristics. Receiver operating characteristic (ROC) curves were constructed to evaluate the discriminatory performance of obesity and metabolic indices for NAFLD in the cross-sectional setting, while time-dependent ROC analysis were applied in the cohort setting to assess prediction accuracy over time. Internal validation was conducted using Bootstrap resampling with 500 iterations. The optimal cut-off values for each index were determined based on the maximum Youden index. Clinical decision curve analysis (DCA) was also performed to evaluate the clinical utility of each model.

All statistical analysis were conducted using R software (version 4.1.0). A two-sided *p* value < 0.05 was considered statistically significant.

## Result

### Baseline characteristics of participants in cross-sectional and cohort studies

Table 1 presents the baseline characteristics of the study population according to NAFLD classification. A total of 789 elderly participants were included, with a mean age of 69.57 years and 43.22% (*n* = 341) were male. Among them, 507 individuals were identified with non-NAFLD, and 282 with NAFLD. Participants with NAFLD were more likely to be male and older (*p* < 0.01), and had higher SBP, weight, WC, BMI, and WHtR (*p* < 0.001). Novel obesity and metabolic indices including LAP, VAI, BRI, CMI, MetS scores, and METS-IR were also significantly higher in the NAFLD group (*p* < 0.01). FBG, TC, and TG levels were elevated in the NAFLD group (*p* < 0.01). No statistically significant differences were observed in FBG, TC, TG, LDL-C, HDL-C, hypertension, or medication use between the groups (*p* > 0.05).

**Table 1:** Baseline characteristics of participants stratified by NAFLD status in the cross-sectional analysis.

Variable	Overall, *N* = 789	Non-NAFLD, *N* = 507	NAFLD, *N* = 282	*p*-value
Gender (*N*,%)				0.003
Male	341.00 (43.22)	239.00 (47.14)	102.00 (36.17)	
Female	448.00 (56.78)	268.00 (52.86)	180.00 (63.83)	
Age (years)	69.57 ± 8.92	68.33 ± 10.02	71.81 ± 5.88	<0.001
SBP (mmHg)	133.37 ± 12.21	132.40 ± 12.49	135.11 ± 11.50	<0.001
DBP (mmHg)	80.03 ± 7.51	79.64 ± 7.63	80.71 ± 7.26	0.074
Height (cm)	158.79 ± 7.99	159.28 ± 8.14	157.91 ± 7.65	0.015
Weight (cm)	61.64 ± 10.22	60.62 ± 10.14	63.49 ± 10.12	<0.001
WC (cm)	88.27 ± 8.74	86.54 ± 8.32	91.40 ± 8.63	<0.001
BMI (kg/m^2^)	24.36 ± 3.06	23.80 ± 2.90	25.38 ± 3.08	<0.001
WHtR	0.56 ± 0.06	0.54 ± 0.05	0.58 ± 0.05	<0.001
METSIR	38.04 (33.91, 43.01)	36.66 (32.95, 41.07)	40.26 (36.31, 44.88)	<0.001
LAP	39.84 (26.46, 62.56)	33.44 (21.43, 50.81)	53.95 (37.45, 79.19)	<0.001
VAI	2.11 (1.29, 3.40)	1.78 (1.14, 2.93)	2.64 (1.81, 4.07)	<0.001
ABSI	0.84 ± 0.06	0.83 ± 0.06	0.84 ± 0.05	<0.001
BRI	4.39 (3.71, 5.22)	4.16 (3.59, 4.87)	4.89 (4.08, 5.84)	<0.001
CMI	1.53 (0.99, 2.56)	1.34 (0.88, 2.11)	2.03 (1.33, 3.09)	<0.001
MetS scores	0.72 (0.26, 1.26)	0.56 (0.15, 1.03)	0.99 (0.52, 1.48)	<0.001
Exercise frequency (*N*,%)				0.5
Daily	711.00 (90.11)	452.00 (89.15)	259.00 (91.84)	
Occasionally	21.00 (2.66)	15.00 (2.96)	6.00 (2.13)	
No exercise	57.00 (7.22)	40.00 (7.89)	17.00 (6.03)	
Smoking status (*N*,%)				0.052
Never	718.00 (91.00)	460.00 (90.73)	258.00 (91.49)	
Former	33.00 (4.18)	17.00 (3.35)	16.00 (5.67)	
Current	38.00 (4.82)	30.00 (5.92)	8.00 (2.84)	
FBG (mmol/L)	7.75 (2.75)	7.64 (2.82)	7.96 (2.62)	0.005
TC (mmol/L)	4.82 (4.14, 5.57)	4.70 (4.01, 5.42)	5.01 (4.35, 5.81)	<0.001
TG (mmol/L)	1.46 (1.06, 2.12)	1.32 (0.98, 1.84)	1.77 (1.32, 2.52)	<0.001
LDL-C (mmol/L)	2.99 (2.30, 3.67)	2.90 (2.25, 3.51)	3.12 (2.43, 3.80)	0.004
HDL-C (mmol/L)	1.18 (1.01, 1.42)	1.19 (1.02, 1.43)	1.17 (0.99, 1.40)	0.11
Hypertension (%)	490.00 (62.10)	309.00 (60.95)	181.00 (64.18)	0.4
Lipid-lowering medication (*N*,%)	12.00 (1.52)	7.00 (1.38)	5.00 (1.77)	0.8
Antihypertensive medication (*N*,%)	373.00 (47.28)	231.00 (45.56)	142.00 (50.35)	0.2
Antidiabetic medication (*N*,%)	438.00 (55.51)	295.00 (58.19)	143.00 (50.71)	0.043

Data are presented as mean ± SD for normally distributed variables, median (interquartile range) for skewed variables, or number (percentage) for categorical variables. Group comparisons between participants with NAFLD and those without NAFLD were performed using Student’s t-test, Mann–Whitney U test, or χ^2^ test, as appropriate. NAFLD, non-alcoholic fatty liver disease; SBP, systolic blood pressure; DBP, diastolic blood pressure; WC, waist circumference; BMI, body mass index; WHtR, waist-to-height ratio; METS-IR, metabolic score for insulin resistance; LAP, lipid accumulation product; VAI, visceral adiposity index; ABSI, a body shape index; BRI, body roundness index; CMI, cardiometabolic index; MetS scores, metabolic syndrome score; FBG, fasting blood glucose; TC, total cholesterol; TG, triglycerides; LDL-C, low-density lipoprotein cholesterol; HDL-C, high-density lipoprotein cholesterol.

In the cohort analysis, a total of 382 participants were included, with a median follow-up duration of 25.37 months. During the follow-up period, 67 individuals developed NAFLD, corresponding to an incidence rate of 8.35 cases per 100 person-years. The mean age of participants was 67.31 years, and 178 (46.6%) were male. Participants who developed NAFLD were more likely to be older, female, and had higher WC, WHtR, TG, and several novel metabolic indices (LAP, VAI, BRI, CMI, MetS scores, METS-IR) compared to those without NAFLD (*p* < 0.05). No significant differences were observed in BMI, SBP, lipid levels, or lifestyle factors. The NAFLD group also had a higher prevalence of hypertension and more frequent use of antidiabetic medications (*p* < 0.05). The specific data can be found in Table 2.

**Table 2:** Baseline characteristics of participants with and without NAFLD in the cohort analysis.

Variable	Overall, *N* = 382	Non-NAFLD, *N* = 315	NAFLD, *N* = 67	*p*-value
Gender (*N*,%)				<0.001
Male	178.00 (46.60)	159.00 (50.48)	19.00 (28.36)	
Female	204.00 (53.40)	156.00 (49.52)	48.00 (71.64)	
Age (years)	67.31 ± 9.98	66.49 ± 10.34	71.16 ± 6.98	<0.001
SBP (mmHg)	131.84 ± 12.34	131.72 ± 12.91	132.37 ± 9.22	0.6
DBP (mmHg)	79.46 ± 7.62	79.42 ± 7.75	79.66 ± 7.06	0.5
Height (cm)	159.47 ± 7.76	160.02 ± 7.78	156.88 ± 7.17	0.002
Weight (cm)	60.52 ± 9.70	60.62 ± 9.67	60.05 ± 9.85	0.4
WC (cm)	86.72 ± 7.99	86.43 ± 7.83	88.11 ± 8.66	0.2
BMI (kg/m^2^)	23.73 ± 2.89	23.60 ± 2.84	24.33 ± 3.08	0.12
WHtR	0.54 ± 0.05	0.54 ± 0.05	0.56 ± 0.06	0.002
METSIR	35.99 (32.93, 40.53)	35.49 (32.89, 39.84)	38.29 (33.87, 43.83)	0.004
LAP	32.85 (21.69, 50.60)	31.44 (21.03, 47.71)	47.06 (31.22, 78.80)	<0.001
VAI	1.70 (1.12, 2.92)	1.57 (1.06, 2.71)	2.72 (1.70, 3.92)	<0.001
ABSI	0.83 ± 0.06	0.83 ± 0.06	0.84 ± 0.06	0.2
BRI	4.24 (3.55, 4.93)	4.08 (3.47, 4.85)	4.46 (3.82, 5.43)	0.002
CMI	1.28 (0.86, 2.08)	1.19 (0.81, 1.82)	1.95 (1.14, 3.17)	<0.001
MetS scores	0.50 (0.11, 0.96)	0.44 (0.06, 0.89)	0.74 (0.31, 1.51)	<0.001
Exercise frequency (*N*,%)				0.9
Daily	357.00 (93.46)	294.00 (93.33)	63.00 (94.03)	
Occasionally	4.00 (1.05)	4.00 (1.27)	0.00 (0.00)	
No exercise	21.00 (5.50)	17.00 (5.40)	4.00 (5.97)	
Smoking status (*N*,%)				0.5
Never	341.00 (89.27)	278.00 (88.25)	63.00 (94.03)	
Former	17.00 (4.45)	15.00 (4.76)	2.00 (2.99)	
Current	24.00 (6.28)	22.00 (6.98)	2.00 (2.99)	
FBG (mmol/L)	6.86 (5.75, 8.60)	6.86 (5.74, 8.57)	6.85 (5.83, 8.60)	0.7
TC (mmol/L)	4.71 (3.95, 5.55)	4.67 (3.95, 5.42)	4.85 (4.22, 5.58)	0.2
TG (mmol/L)	1.31 (0.97, 1.85)	1.24 (0.93, 1.70)	1.76 (1.28, 2.53)	<0.001
LDL-C (mmol/L)	2.97 (2.27, 3.62)	2.97 (2.21, 3.57)	2.86 (2.38, 3.68)	0.7
HDL-C (mmol/L)	1.24 (1.05, 1.48)	1.26 (1.07, 1.51)	1.13 (1.01, 1.35)	0.009
Hypertension (*N*,%)	225.00 (58.90)	175.00 (55.56)	50.00 (74.63)	0.004
Lipid-lowering medication (*N*,%)	4.00 (1.05)	4.00 (1.27)	0.00 (0.00)	>0.9
Antihypertensive medication (*N*,%)	178.00 (46.60)	138.00 (43.81)	40.00 (59.70)	0.018
Antidiabetic medication (*N*,%)	261.00 (68.32)	213.00 (67.62)	48.00 (71.64)	0.5

Data are expressed as mean ± SD for normally distributed variables, median (interquartile range) for skewed variables, or number (percentage) for categorical variables. Comparisons between NAFLD and non-NAFLD groups were conducted using Student’s t-test, Mann–Whitney U test, or χ^2^ test, as appropriate. NAFLD, non-alcoholic fatty liver disease; SBP, systolic blood pressure; DBP, diastolic blood pressure; WC, waist circumference; BMI, body mass index; WHtR, waist-to-height ratio; METS-IR, metabolic score for insulin resistance; LAP, lipid accumulation product; VAI, visceral adiposity index; ABSI, a body shape index; BRI, body roundness index; CMI, cardiometabolic index; MetS scores, metabolic syndrome score; FBG, fasting blood glucose; TC, total cholesterol; TG, triglycerides; LDL-C, low-density lipoprotein cholesterol; HDL-C, high-density lipoprotein cholesterol.

### Independent associations of obesity- and metabolism-related indices with NAFLD in cross-sectional analysis

To assess the independent effect of various obesity and metabolic indices and the risk of NAFLD, logistic regression analysis was conducted using both continuous variables and quartile-based groupings. As shown in Table 3, all obesity and metabolic indices were associated with increased risk of NAFLD in both unadjusted (Model 1) and adjusted (Model 2) models. Compared with individuals in the lowest quartile (Q1), those in the highest quartile (Q4) had the following OR for NAFLD in the fully adjusted model (Model 2): LAP (OR = 13.20, 95% CI: 7.55–24.14), METS-IR (OR = 6.87, 95% CI: 4.18–11.51), VAI (OR = 5.55, 95% CI: 3.41–9.19), BRI (OR = 5.82, 95% CI: 3.62–9.54), CMI (OR = 5.88, 95% CI: 3.82–9.99), MetS scores (OR = 5.16, 95% CI: 3.22–8.42), BMI (OR = 3.82, 95% CI: 2.69–5.49) and WHtR (OR = 5.68, 95% CI: 3.60–8.96) In addition, the presence of MS was independently associated with a higher risk of NAFLD (OR = 3.14, 95% CI: 2.31–4.70), supporting the role of metabolic dysfunction in NAFLD pathogenesis.

**Table 3:** Association between metabolic indices and NAFLD risk in binary logistic model.

	Model 1			Model 2		
Variable	OR	95%CI	*P*	OR	95%CI	*P*
BMI (continuity)	1.193	1.133, 1.257	<0.001	1.246	1.178, 1.322	<0.001
BMI (Q1)	Reference			Reference		
BMI (Q2)	1.47	1.018, 2.118	0.039	1.758	1.194, 2.587	0.004
BMI (Q3)	2.881	2.001, 4.163	<0.001	3.735	2.516, 5.585	<0.001
BMI (Q4)	5.333	1.851, 17.45	0.003	8.318	2.687, 29.148	<0.001
WHtR (continuity)	3.526	2.621, 4.802	<0.001	3.288	2.414, 4.533	<0.001
WHtR (Q1)	Reference			Reference		
WHtR (Q2)	2.006	1.277, 3.169	0.003	1.983	1.25, 3.164	0.004
WHtR (Q3)	2.797	1.814, 4.357	<0.001	2.624	1.681, 4.136	<0.001
WHtR (Q4)	6.251	4.044, 9.804	<0.001	5.677	3.595, 9.092	<0.001
METS-IR (continuity)	1.087	1.062, 1.113	<0.001	1.113	1.085, 1.143	<0.001
METS-IR (Q1)	Reference			Reference		
METS-IR (Q2)	2.004	1.258, 3.23	0.004	2.28	1.401, 3.756	0.001
METS-IR (Q3)	3.486	2.221, 5.561	<0.001	4.315	2.679, 7.073	<0.001
METS-IR (Q4)	4.593	2.931, 7.323	<0.001	6.867	4.184, 11.51	<0.001
LAP (continuity)	1.013	1.009, 1.017	<0.001	1.014	1.01, 1.018	<0.001
LAP (Q1)	Reference			Reference		
LAP (Q2)	4.639	2.702, 8.298	<0.001	4.882	2.805, 8.844	<0.001
LAP (Q3)	7.924	4.664, 14.074	<0.001	8.041	4.634, 14.568	<0.001
LAP (Q4)	11.432	6.733, 20.318	<0.001	13.196	7.546, 24.139	<0.001
VAI (continuity)	1.052	1.012, 1.101	0.02	1.049	1.011, 1.098	0.027
VAI (Q1)	Reference			Reference		
VAI (Q2)	2.299	1.437, 3.728	0.001	2.323	1.426, 3.836	0.001
VAI (Q3)	3.576	2.265, 5.745	<0.001	3.402	2.11, 5.577	<0.001
VAI (Q4)	5.159	3.275, 8.288	<0.001	5.545	3.414, 9.186	<0.001
ABSI (continuity)	1.538	1.18, 2.014	0.002	1.301	0.981, 1.731	0.069
ABSI (Q1)	Reference			Reference		
ABSI (Q2)	1.543	1.012, 2.356	0.044	1.444	0.934, 2.234	0.098
ABSI (Q3)	1.641	1.093, 2.473	0.017	1.412	0.923, 2.164	0.112
ABSI (Q4)	1.903	1.264, 2.877	0.002	1.503	0.974, 2.324	0.066
BRI (continuity)	1.777	1.55, 2.049	<0.001	1.721	1.492, 1.996	<0.001
BRI (Q1)	Reference			Reference		
BRI (Q2)	1.878	1.174, 3.035	0.009	1.865	1.153, 3.05	0.012
BRI (Q3)	2.664	1.689, 4.261	<0.001	2.475	1.545, 4.017	<0.001
BRI (Q4)	6.333	4.033, 10.135	<0.001	5.822	3.62, 9.536	<0.001
CMI (continuity)	1.096	1.034, 1.169	0.004	1.107	1.042, 1.185	0.002
CMI (Q1)	Reference			Reference		
CMI (Q2)	2.25	1.411, 3.636	0.001	2.362	1.461, 3.867	0.001
CMI (Q3)	3.373	2.143, 5.398	<0.001	3.242	2.031, 5.258	<0.001
CMI (Q4)	5.065	3.223, 8.109	<0.001	6.085	3.782, 9.987	<0.001
MetS scores (continuity)	1.442	1.246, 1.683	<0.001	1.491	1.28, 1.754	<0.001
MetS scores (Q1)	Reference			Reference		
MetS scores (Q2)	1.732	1.079, 2.808	0.024	1.891	1.162, 3.108	0.011
MetS scores (Q3)	3.999	2.551, 6.375	<0.001	4.255	2.672, 6.891	<0.001
MetS scores (Q4)	4.427	2.826, 7.057	<0.001	5.159	3.219, 8.421	<0.001
MetS	2.48	1.798, 3.451	<0.001	3.144	2.131, 4.703	<0.001

Odds ratio (OR) and 95% confidence intervals (CIs) were calculated for both continuous and quartile-based forms of each metabolic index. Model 1: the unadjusted logistic regression model. Model 2: adjusted for sex, age, exercise frequency, smoking status, antihypertensive medication, antidiabetic medication, lipid-lowering medication, and hypertension. BMI, body mass index; WHtR, waist-to-height ratio; METS-IR, metabolic score for insulin resistance; LAP, lipid accumulation product; VAI, visceral adiposity index; ABSI, a body shape index; BRI, body roundness index; CMI, cardiometabolic index; MetS scores, metabolic syndrome score; MetS, metabolic syndrome.

**Figure 2:**
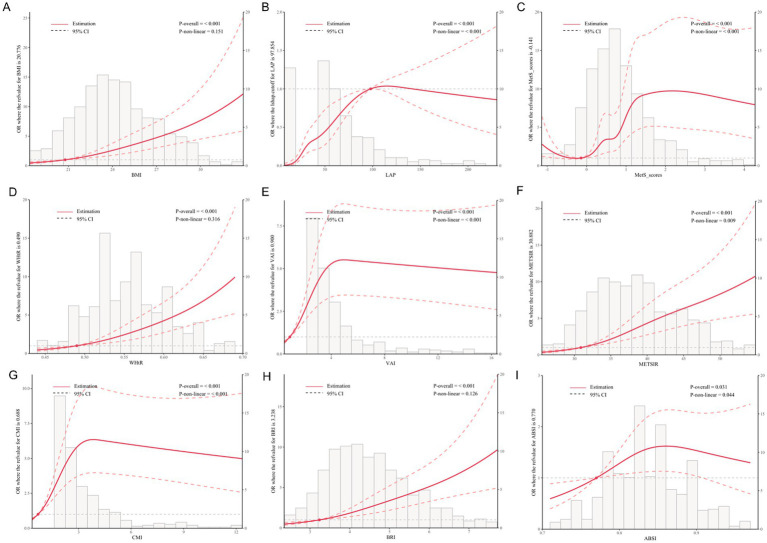
Dose–response relationships between metabolic indices and NAFLD risk based on restricted cubic spline analysis. The solid red line indicates the estimated OR, and the dashed lines represent the 95% confidence intervals. *p*-values for overall and non-linear associations are provided. Histograms indicate the distribution of each index in the study population. **(A)** BMI, **(B)** LAP, **(C)** MetS scores, **(D)** WHtR, **(E)** VAI, **(F)** METS-IR, **(G)** CMI, **(H)** BRI, **(I)** ABSI. All models were adjusted for sex, age, exercise frequency, smoking status, antihypertensive medication, antidiabetic medication, lipid-lowering medication, and hypertension.

Figure 2 presents the dose–response relationships between obesity and metabolic indices and NAFLD risk. The overall associations were statistically significant for all indicators (*P*-overall < 0.05). Nonlinear relationships were observed for LAP (*P*-nonlinear < 0.001), MetS scores (*P*-nonlinear < 0.001), VAI (*P*-nonlinear < 0.001), CMI (*P*-nonlinear < 0.001), METS-IR (*P*-nonlinear = 0.009), and ABSI (*P*-nonlinear = 0.044).

Subgroup analysis examining the associations between obesity measures and NAFLD was conducted across eight categories, including gender, age, abdominal obesity, overweight, hypertension, elevated TG, reduced HDL-C, and MetS. Statistically significant positive associations between WHtR, METS-IR, BRI, and BMI with NAFLD were observed across all subgroups. Similar trends were found in most subgroups, indicating the robustness of these associations. Several significant interactions were identified. Details of the subgroup-specific estimates and interaction terms are presented in Supplementary Figures S2–S10.

### Obesity- and metabolism-related indices identified as predictors of increased NAFLD risk in longitudinal analysis

**Figure 3:**
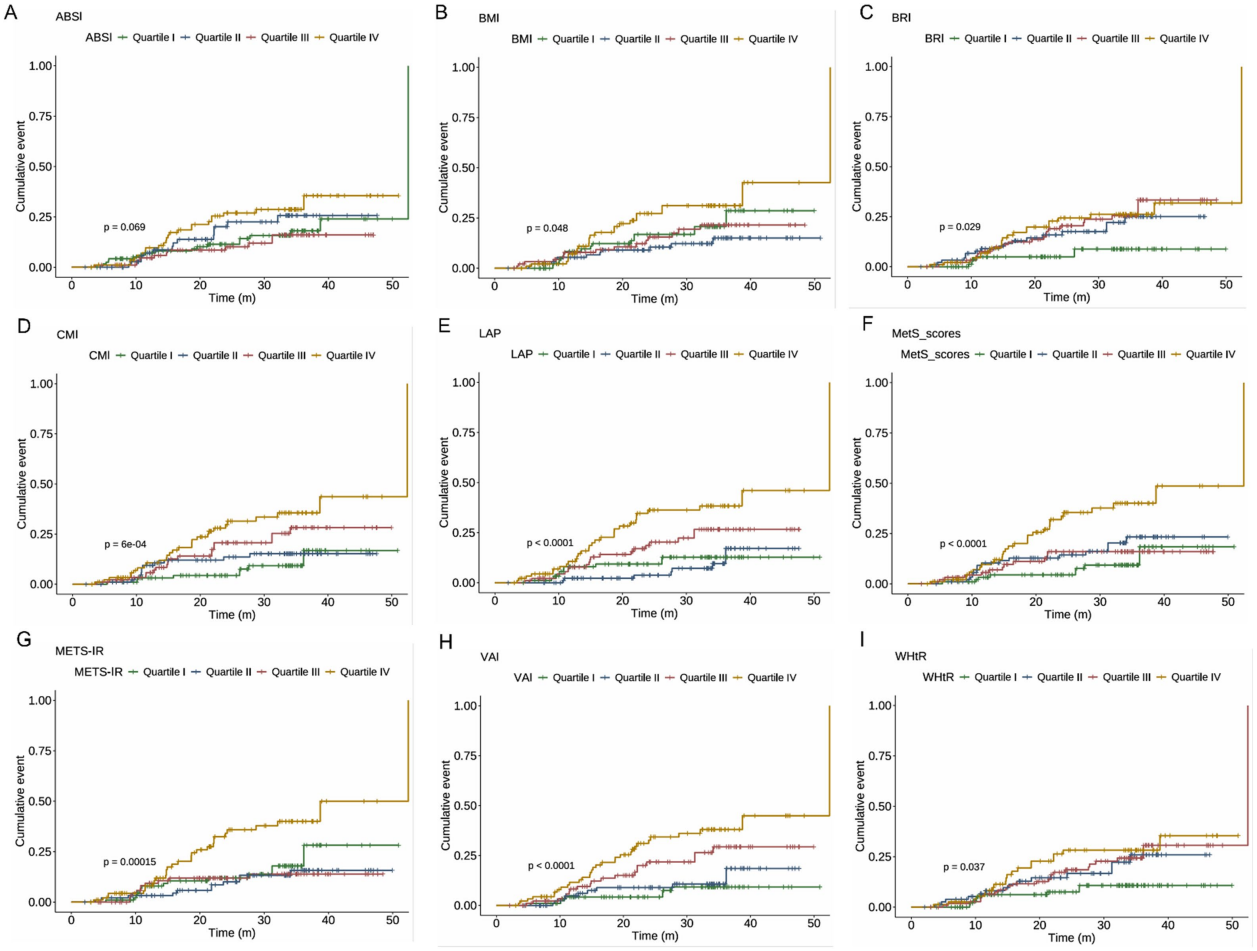
c for the cumulative incidence of NAFLD across quartiles of metabolic indices. Log-rank test *p*-values are presented in each panel to indicate statistical differences among quartiles. **(A)** ABSI, **(B)** BMI, **(C)** BRI, **(D)** CMI, **(E)** LAP, **(F)** MetS scores, **(G)** METS-IR, **(H)** VAI, and **(I)** WHtR. NAFLD, non-alcoholic fatty liver disease; ABSI, a body shape index; BMI, body mass index; BRI, body roundness index; CMI, cardiometabolic index; LAP, lipid accumulation product; METS-IR, metabolic score for insulin resistance; VAI, visceral adiposity index; WHtR, waist-to-height ratio.

Cumulative incidence curves were compared across quartiles of obesity indicators. As shown in Figure 3, including LAP, METS-IR, MetS scores, VAI, CMI, BMI, BRI, and WHtR were significantly associated with NAFLD incidence, with higher quartiles showing greater risk (all log-rank test *p* < 0.05). ABSI showed a non-significant trend (*p* = 0.069).

After adjustment for confounding factors, Cox proportional hazards models showed significant associations between incident NAFLD and METS-IR (HR = 2.784, 95% CI: 1.465, 5.292, *p* = 0.002), LAP (HR = 3.104, 95% CI: 1.481, 6.505, *p* = 0.003), and MetS scores (HR = 4.256, 95% CI: 1.989, 9.107, *p* < 0.001). No significant associations were observed for WHtR, ABSI, or BRI (all *p* > 0.05). The HR for incident NAFLD per one standard deviation increase in BMI was 1.114 (95% CI: 1.020, 1.217, *p* = 0.017). Compared with the non-MetS group, the MetS group had a 1.912-fold increased risk of incident NAFLD (95% CI: 1.018, 3.591). Further details are provided in Table 4.

**Table 4:** Associations between metabolic indices and the risk of NAFLD based on Cox proportional hazards models.

	Model 1			Model 2		
Variable	HR	95%CI	*P*	HR	95%CI	*P*
BMI (continuity)	1.093	1.003, 1.19	0.043	1.114	1.02, 1.217	0.017
BMI (Q1)	Reference					
BMI (Q2)	0.578	0.27, 1.233	0.156	0.633	0.295, 1.359	0.241
BMI (Q3)	0.884	0.441, 1.769	0.727	0.94	0.466, 1.896	0.863
BMI (Q4)	1.523	0.813, 2.854	0.189	1.68	0.876, 3.221	0.118
WHtR (continuity)	2.152	1.312, 3.528	0.002	1.581	0.94, 2.659	0.084
WHtR (Q1)	Reference					
WHtR (Q2)	2.242	0.97, 5.18	0.059	1.971	0.839, 4.629	0.119
WHtR (Q3)	2.433	1.131, 5.235	0.023	2.061	0.948, 4.478	0.068
WHtR (Q4)	3.06	1.384, 6.764	0.006	2.024	0.877, 4.671	0.098
METS-IR (continuity)	1.069	1.031, 1.109	<0.001	1.085	1.043, 1.129	<0.001
METS-IR (Q1)	Reference					
METS-IR (Q2)	0.675	0.31, 1.471	0.323	0.836	0.38, 1.84	0.657
METS-IR (Q3)	0.737	0.338, 1.605	0.442	0.872	0.393, 1.932	0.736
METS-IR (Q4)	2.284	1.224, 4.263	0.009	2.784	1.465, 5.292	0.002
LAP (continuity)	1.008	1.005, 1.011	<0.001	1.008	1.004, 1.012	<0.001
LAP (Q1)	Reference					
LAP (Q2)	0.73	0.288, 1.851	0.508	0.705	0.275, 1.804	0.466
LAP (Q3)	1.952	0.908, 4.2	0.087	1.542	0.705, 3.373	0.278
LAP (Q4)	3.515	1.712, 7.216	0.001	3.102	1.481, 6.5	0.003
VAI (continuity)	1.019	0.996, 1.042	0.1	1.015	0.99, 1.04	0.239
VAI (Q1)	Reference					
VAI (Q2)	1.6	0.609, 4.205	0.34	1.547	0.578, 4.139	0.385
VAI (Q3)	3.288	1.382, 7.824	0.007	2.801	1.147, 6.844	0.024
VAI (Q4)	5.401	2.371, 12.301	<0.001	4.843	2.058, 11.397	<0.001
ABSI (continuity)	1.159	0.77, 1.743	0.479	0.898	0.583, 1.382	0.624
ABSI (Q1)	Reference					
ABSI (Q2)	1.379	0.679, 2.8	0.374	1.207	0.583, 2.495	0.613
ABSI (Q3)	0.802	0.376, 1.712	0.568	0.665	0.305, 1.451	0.305
ABSI (Q4)	1.857	0.997, 3.457	0.051	1.357	0.694, 2.653	0.372
BRI (continuity)	1.434	1.146, 1.796	0.002	1.241	0.976, 1.578	0.078
BRI (Q1)	Reference					
BRI (Q2)	2.895	1.141, 7.344	0.025	2.665	1.033, 6.871	0.043
BRI (Q3)	3.394	1.376, 8.373	0.008	2.899	1.162, 7.233	0.023
BRI (Q4)	3.479	1.404, 8.62	0.007	2.407	0.947, 6.115	0.065
CMI (continuity)	1.036	1, 1.074	0.048	1.031	0.993, 1.07	0.109
CMI (Q1)	Reference					
CMI (Q2)	1.537	0.648, 3.649	0.33	1.64	0.685, 3.922	0.267
CMI (Q3)	2.512	1.128, 5.593	0.024	2.197	0.973, 4.96	0.058
CMI (Q4)	3.911	1.838, 8.322	<0.001	3.951	1.836, 8.502	<0.001
MetS scores (continuity)	1.311	1.153, 1.49	<0.001	1.308	1.132, 1.512	<0.001
MetS scores (Q1)	Reference					
MetS scores (Q2)	1.97	0.87, 4.459	0.104	1.98	0.872, 4.494	0.102
MetS scores (Q3)	1.549	0.652, 3.678	0.321	1.509	0.63, 3.615	0.356
MetS scores (Q4)	4.263	2.015, 9.018	<0.001	4.256	1.989, 9.107	<0.001
MetS	2.275	1.31, 3.951	0.004	1.912	1.018, 3.591	0.044

Hazard ratio (HR), 95% confidence intervals (CIs) were calculated for both continuous and quartile-based forms of each metabolic index. Model 1: the unadjusted model. Model 2: adjusted for sex, age, exercise frequency, smoking status, antihypertensive medication, antidiabetic medication, lipid-lowering medication, and hypertension. BMI, body mass index; WHtR, waist-to-height ratio; METS-IR, metabolic score for insulin resistance; LAP, lipid accumulation product; VAI, visceral adiposity index; ABSI, a body shape index; BRI, body roundness index; CMI, cardiometabolic index; MetS scores, metabolic syndrome score; MetS, metabolic syndrome.

**Figure 4:**
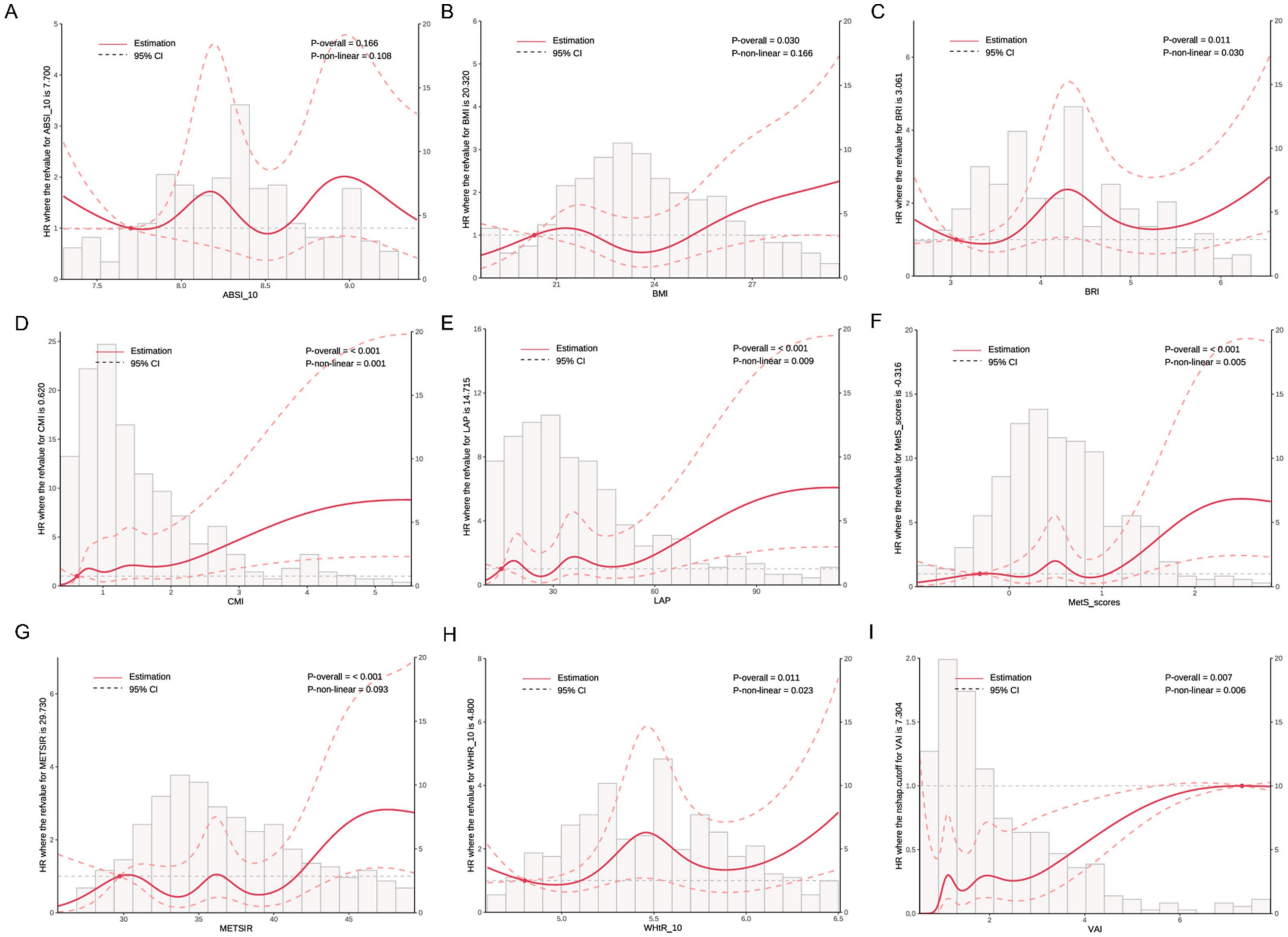
Dose–response relationships between metabolic indices and NAFLD incidence based on restricted cubic spline analysis. Solid red lines represent the estimated HRs, and dashed lines represent the 95% confidence intervals (CIs). Histograms show the distribution of each index in the study population. *p*-values for overall and non-linear associations are provided in each panel. **(A)** ABSI, **(B)** BMI, **(C)** BRI, **(D)** CMI, **(E)** LAP, **(F)** MetS scores, **(G)** METS-IR, **(H)** WHtR, and **(I)** VAI. All models were adjusted for sex, age, exercise frequency, smoking status, antihypertensive medication, antidiabetic medication, lipid-lowering medication, and hypertension.

As shown in Figure 4, RCS analysis revealed dose–response relationships between obesity indicators and the risk of incident NAFLD. Significant linear associations were observed for CMI (*P*-overall < 0.001, *P*-nonlinear = 0.001), LAP (*P*-overall < 0.001, *P*-nonlinear = 0.009), MetS scores (*P*-overall < 0.001, *P*-nonlinear = 0.005), and METS-IR (*P*-overall < 0.001, *P*-nonlinear = 0.003). Nonlinear associations were found for BRI (*P*-nonlinear = 0.030), WHtR (*P*-nonlinear = 0.023), and VAI (*P*-nonlinear = 0.006). BMI showed a borderline linear association (*P*-overall = 0.030), while ABSI did not show a significant trend (*P*-overall = 0.166).

Stratified analysis revealed notable variations in the associations between obesity and metabolic indices and NAFLD across subgroups. BMI was specifically associated with NAFLD risk in females, while BRI, CMI, MetS scores, VAI, and WHtR consistently exhibited stronger associations in males. METS-IR showed associations across nearly all subgroups, indicating consistent predictive value. In contrast, ABSI showed no significant associations in any subgroup. The results further indicated that VAI significantly interacted with all stratifying variables (all *P*-interaction < 0.05), suggesting potential effect modification. Detailed subgroup-specific results are presented in Supplementary Figures S11–S19.

### Obesity- and metabolism-related indices demonstrate stable predictive accuracy for NAFLD

The predictive performance of nine indicators for NAFLD was evaluated using both cross-sectional and time-dependent ROC analysis. In the cross-sectional analysis (Supplementary Figure S20), LAP exhibited the highest AUC (0.72, 95% CI: 0.68–0.76) for predicting NAFLD, followed by WHtR (0.684, 95% CI: 0.646–0.722), BRI (0.684, 95% CI: 0.645–0.722), CMI (0.664, 95% CI: 0.626–0.702). Based on the time-dependent ROC analysis shown in Supplementary Figure S21, the predictive performance of obesity-related indicators for incident NAFLD varied over time (12, 24, and 36 months). LAP consistently demonstrated the highest AUCs at all time points—0.565 (95% CI: 0.435–0.696) at 12 months, increasing to 0.725 (0.645–0.805) at 24 months, and slightly declining to 0.671 (95% CI: 0.580–0.761) at 36 months. Other indicators, including CMI (AUC = 0.597 to 0.689), VAI (AUC = 0.622 to 0.698), and MetS scores (AUC = 0.592 to 0.655), also showed favorable predictive values, particularly at 24 months. After internal validation using Bootstrap resampling, the predictive performance of the obesity-related indicators remained largely stable (Supplementary Table S1). LAP still achieved the highest corrected AUCs at all time points—0.563 (95% CI: 0.434–0.687) at 12 months, 0.726 (95% CI: 0.650–0.812) at 24 months, and 0.670 (95% CI: 0.586–0.768) at 36 months. Similarly, the corrected AUCs for CMI, VAI, and MetS scores at 24 months were 0.725 (95% CI: 0.650–0.804), 0.734 (95% CI: 0.667–0.804), and 0.722 (95% CI: 0.652–0.797), respectively, confirming their favorable predictive value.

### Obesity- and metabolism-related indices provide net clinical benefit with optimal cut-off values established

The DCA curve showed that all indicators provided net benefits over “treat all” and “treat none” strategies, especially at lower thresholds, with LAP, VAI, and MetS scores performing best (Supplementary Figure S22). Optimal cut-off values for the nine indicators were determined based on the maximum Youden index (Supplementary Table S2), offering reference thresholds for clinical application.

## Discussion

Our study adds new insights by comprehensively evaluating obesity- and metabolism-related indices in relation to NAFLD using both cross-sectional and longitudinal cohort designs. In the cross-sectional analysis, all examined indices were found to be significantly associated with the presence of NAFLD, including BMI, WHtR, LAP, VAI, ABSI, BRI, CMI, METS-IR, and MetS scores. In contrast, in the longitudinal cohort analysis, WHtR, VAI, ABSI, BRI, and CMI did not retain statistical significance after adjustment for relevant covariates. When both the magnitude of association and discriminatory performance were taken into account, LAP and MetS scores emerged as the most robust predictors. Moreover, MetS consistently remained an independent risk factor for NAFLD across both analytic approaches. The identification of high-risk NAFLD patients among elderly individuals with type 2 diabetes in primary care settings may be facilitated by the incorporation of these indices into routine clinical practice.

Our findings highlight that multiple indicators reflecting metabolic abnormalities, as well as the presence of MetS, are significantly associated with NAFLD risk in individuals with T2DM. These indices, which integrate lipid metabolism, glycemic status, and fat distribution, effectively capture the overall metabolic burden. Similar associations have been reported in previous studies, where BMI, WHtR, METS-IR, LAP, VAI, ABSI, BRI, and CMI have all been used to predict NAFLD risk (23–27). TyG-BMI effectively predicts NAFLD comorbidity in T2DM patients because it integrates information on glucose metabolism, lipid status, and obesity (28). Although the predictive value of these obesity and metabolic indices has been established in general populations, systematic evaluations targeting elderly T2DM patients—who represent a high-risk subgroup—remain limited. This population is typically characterized by more severe glycemic-lipidemic dysregulation and a higher prevalence of MetS, which may lead to distinct clinical presentations and progression patterns of NAFLD. At the pathophysiological level, multiple mechanisms have been implicated in the development of NAFLD under metabolic dysregulation. Specifically, adipose tissue dysfunction increases FFA release, which promotes TG synthesis upon hepatic uptake. Concurrently, impaired insulin signaling suppresses FA *β*-oxidation and VLDL secretion, exacerbating hepatic lipid accumulation (29). Moreover, IR enhances hepatic steatosis through the activation of lipogenic transcription factors (30, 31). Chronic low-grade inflammation and oxidative stress further induce Kupffer cell activation and pro-inflammatory cytokine release, accelerating hepatocyte injury and fibrosis progression (32). Together, these mechanisms provide a biological foundation for the increased susceptibility to NAFLD in T2DM patients and support the predictive utility of the obesity and metabolic indices observed in this study.

LAP and MetS score emerged as the most effective predictors of NAFLD in this study, showing independent associations and achieving AUCs exceeding 0.70 at the 24-month follow-up. LAP, calculated from WC and fasting TG levels, integrates markers of visceral adiposity and dyslipidemia, and has been recognized as a sensitive proxy for adipose tissue dysfunction (33). Its relevance to NAFLD has been consistently demonstrated in prior studies. For example, a cross-sectional analysis of U.S. adults identified LAP as a key indicator for NAFLD and MAFLD risk (34), while a meta-analysis confirmed its potential value for NAFLD screening (35). In elderly populations, LAP has been closely linked to NAFLD occurrence and proposed as a useful tool for screening and management (36). By incorporating both cross-sectional and longitudinal analysis, our study provides additional evidence supporting the predictive stability of LAP for NAFLD over time.

MetS score is a metabolic syndrome index specifically developed for the Chinese population, quantifying the clustering of hyperglycemia, central obesity, hypertension, hypertriglyceridemia, and low HDL-C (17). It reflects systemic IR, lipotoxicity, and low-grade inflammation, while avoiding information loss associated with traditional binary definitions, thus enabling a more accurate assessment of metabolic disturbance severity (37). Existing studies have only explored its association with frailty progression in older adults (38). In T2DM patients, where decreased insulin sensitivity and increased metabolic load are common, the MetS score exhibited stable and significant predictive performance for NAFLD in this study.

Subgroup analysis showed that MetS score and LAP had stronger predictive value for NAFLD in specific T2DM subpopulations, especially among males, individuals over 70 years, those who were overweight, centrally obese, hypertensive, or had low HDL-C levels. These groups tend to have more severe metabolic dysfunction and higher visceral fat and insulin resistance, which may accelerate NAFLD development. A significant interaction between sex and age was also observed, indicating that male and older patients may be more responsive to risk prediction using MetS score and LAP. These findings support a more individualized approach to NAFLD screening in elderly patients with T2DM.

In this study, we provide a set of optimal cut-off values for obesity- and metabolism-related indices in elderly patients with type 2 diabetes, offering new evidence for clinical practice. Although previous studies have also reported some reference cut-off values, they were derived from the general population (39, 40). Therefore, the cut-off values identified in our study are more applicable to elderly patients with T2DM. Furthermore, decision curve analysis showed the good clinical utility of these indices in predicting NAFLD risk, offering clinicians practical tools for risk stratification and early intervention.

This study has several notable strengths that enhance the reliability and applicability of its findings. By integrating both cross-sectional and prospective cohort designs, the associations between nine obesity and metabolic indices and NAFLD were evaluated from complementary perspectives, which strengthens the robustness of the conclusions. Moreover, the study population consisted of elderly individuals with T2DM, who are known to exhibit a high degree of metabolic dysregulation, thereby ensuring that the findings are clinically relevant and tailored to a high-risk population. Another important strength lies in the practicality of the selected indices, as all were derived from routine anthropometric and biochemical parameters, without the need for advanced imaging techniques or specialized equipment, making them particularly suitable for implementation in resource-limited or primary care settings. Among the indices assessed, MetS score and VAI were identified as the most effective in predicting NAFLD, highlighting their potential utility in early risk screening.

However, several limitations should be acknowledged. First, the follow-up duration in the cohort analysis was relatively short and the sample size was limited, resulting in a small number of incident NAFLD cases, which may have reduced the statistical power to detect associations; nevertheless, the sample size in this study met the minimum requirement. Second, as a single-center study conducted within a specific geographic region, the generalizability of the findings remains to be verified; however, internal validation confirmed the robustness of the results. Third, although major confounders were adjusted for, residual confounding from unmeasured factors, such as dietary habits, specific types and intensity of physical activity, the effects of different antidiabetic medications, and the duration of diabetes, cannot be entirely ruled out. More potential confounders should be investigated in future studies to further reduce bias. Fourth, NAFLD was diagnosed solely based on ultrasound, which, although widely used in clinical practice, may have limited sensitivity and accuracy compared to more quantitative imaging modalities. Future studies with longer follow-up periods, larger sample sizes, multi-center designs, and more precise diagnostic methods are warranted to validate these findings and further elucidate the underlying mechanisms.

## Conclusion

MetS score and LAP were found to be reliable and practical indicators for predicting NAFLD in elderly individuals with T2DM. Their strong predictive performance and ease of use make them particularly suitable for application in primary healthcare settings. As low-cost and non-invasive tools, they offer a feasible approach for early risk assessment and targeted intervention. These findings highlight the importance of addressing metabolic abnormalities as a key strategy in preventing NAFLD among older adults with T2DM and support the integration of these indices into routine screening practices to improve disease management at the community level.

## Data Availability

Due to ethical and privacy concerns, the data are not publicly available but can be requested from the corresponding author.
