# Supplementary material for: Novel obesity and metabolic indices better predict non-alcoholic fatty liver disease in elderly T2DM patients: evidence from cross-sectional and longitudinal analysis
Source: Front Med (Lausanne). 2025 Sep 22;12:1649466. doi: 10.3389/fmed.2025.1649466 (PMC12497769; doi:10.3389/fmed.2025.1649466)
Supplement: Supplementary file 1 [file Table_1.DOCX]

Table S1 Time-dependent ROC AUCs and 95% Confidence Intervals After Bootstrap Internal Validation

| Variable | 12 Months | |  | 24 Months | |  | 36 Months | |
| --- | --- | --- | --- | --- | --- | --- | --- | --- |
|  | AUC | 95%CI |  | AUC | 95%CI |  | AUC | 95%CI |
| BMI | 0.464 | 0.344, 0.585 |  | 0.588 | 0.492, 0.674 |  | 0.563 | 0.464, 0.648 |
| WHtR | 0.506 | 0.379, 0.631 |  | 0.636 | 0.557, 0.725 |  | 0.566 | 0.471, 0.662 |
| METS-IR | 0.529 | 0.41, 0.651 |  | 0.67 | 0.59, 0.752 |  | 0.617 | 0.522, 0.704 |
| LAP | 0.563 | 0.434, 0.687 |  | 0.726 | 0.65, 0.812 |  | 0.67 | 0.586, 0.768 |
| VAI | 0.62 | 0.519, 0.72 |  | 0.734 | 0.667, 0.804 |  | 0.699 | 0.615, 0.785 |
| ABSI | 0.5 | 0.384, 0.614 |  | 0.538 | 0.461, 0.61 |  | 0.493 | 0.412, 0.574 |
| BRI | 0.498 | 0.37, 0.621 |  | 0.634 | 0.553, 0.723 |  | 0.568 | 0.471, 0.662 |
| CMI | 0.595 | 0.486, 0.702 |  | 0.725 | 0.656, 0.804 |  | 0.69 | 0.606, 0.779 |
| MetS scores | 0.589 | 0.475, 0.703 |  | 0.722 | 0.652, 0.797 |  | 0.653 | 0.559, 0.739 |

Table S2 Optimal Cut-off Values of Nine Obesity- and Metabolism-Related Indices

| Variable | cut-off | sensitivity | specificity | Youden Index |
| --- | --- | --- | --- | --- |
| BMI | 24.915 | 0.433 | 0.717 | 0.15 |
| WHtR | 0.535 | 0.716 | 0.476 | 0.193 |
| METS-IR | 40.5 | 0.463 | 0.794 | 0.256 |
| LAP | 35.52 | 0.716 | 0.59 | 0.307 |
| VAI | 1.795 | 0.746 | 0.584 | 0.33 |
| ABSI | 0.875 | 0.358 | 0.794 | 0.152 |
| BRI | 4.145 | 0.716 | 0.517 | 0.234 |
| CMI | 1.945 | 0.507 | 0.778 | 0.285 |
| MetS scores | 1.308 | 0.373 | 0.902 | 0.275 |

Table S3 Abbreviations Used in This Study

| Abbreviation | Full Term |
| --- | --- |
| NAFLD | Non-alcoholic fatty liver disease |
| T2DM | Type 2 diabetes mellitus |
| MetS | Metabolic syndrome |
| MetS score | Metabolic syndrome score |
| LAP | Lipid accumulation product |
| VAI | Visceral adiposity index |
| ABSI | A body shape index |
| BRI | Body roundness index |
| CMI | Cardiometabolic index |
| METS-IR | Metabolic score for insulin resistance |
| BMI | Body mass index |
| WC | Waist circumference |
| WHtR | Waist-to-height ratio |
| SBP | Systolic blood pressure |
| DBP | Diastolic blood pressure |
| FBG | Fasting blood glucose |
| FPG | Fasting plasma glucose |
| TG | Triglycerides |
| TC | Total cholesterol |
| HDL-C | High-density lipoprotein cholesterol |
| LDL-C | Low-density lipoprotein cholesterol |
| MAP | Mean arterial pressure |
| OR | Odds ratio |
| HR | Hazard ratio |
| CI | Confidence interval |
| RCS | Restricted cubic spline |
| ROC | Receiver operating characteristic |
| AUC | Area under the curve |
| DCA | Decision curve analysis |
| DAG | Directed acyclic graph |
| CDS | Chinese Diabetes Society |
| BPHS | Basic Public Health Service |
| IR | Insulin resistance |


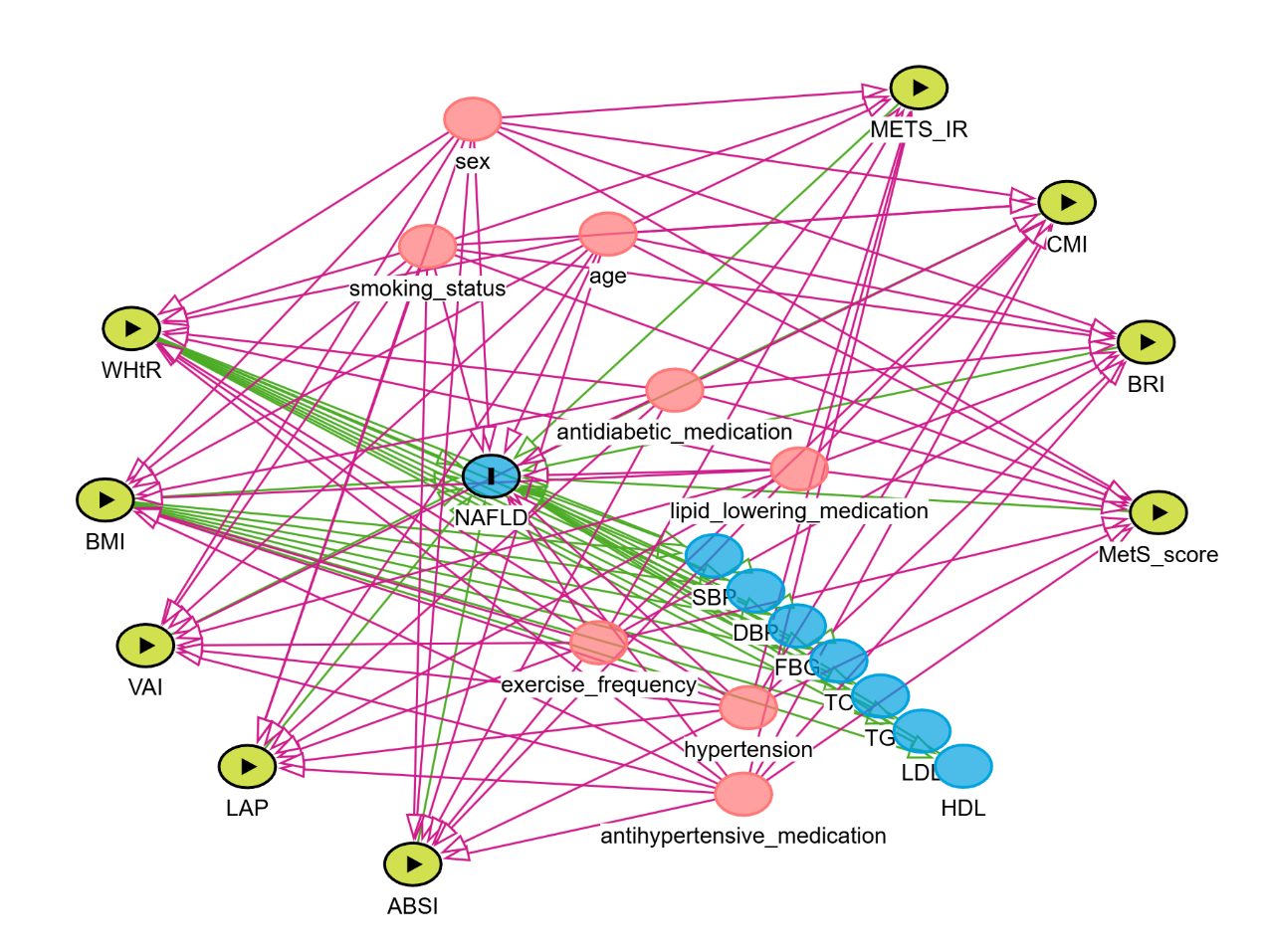


Figure S1 Directed acyclic graph of study variables and NAFLD


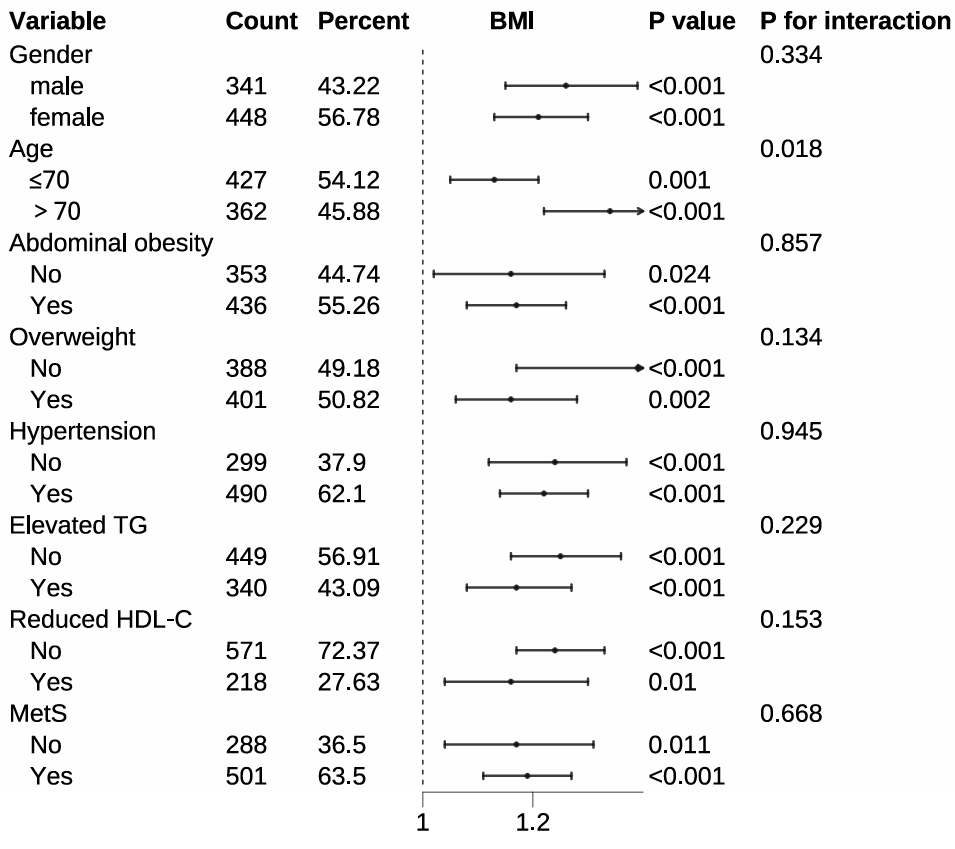


Figure S2 Subgroup analysis of the association between BMI and NAFLD risk in cross-sectional studies

All models were adjusted for sex, age, exercise frequency, smoking status, antihypertensive medication, antidiabetic medication, lipid-lowering medication, and hypertension, except for the stratification variable.

BMI, body mass index; TG, triglycerides; HDL-C, high-density lipoprotein cholesterol; MetS, metabolic syndrome.


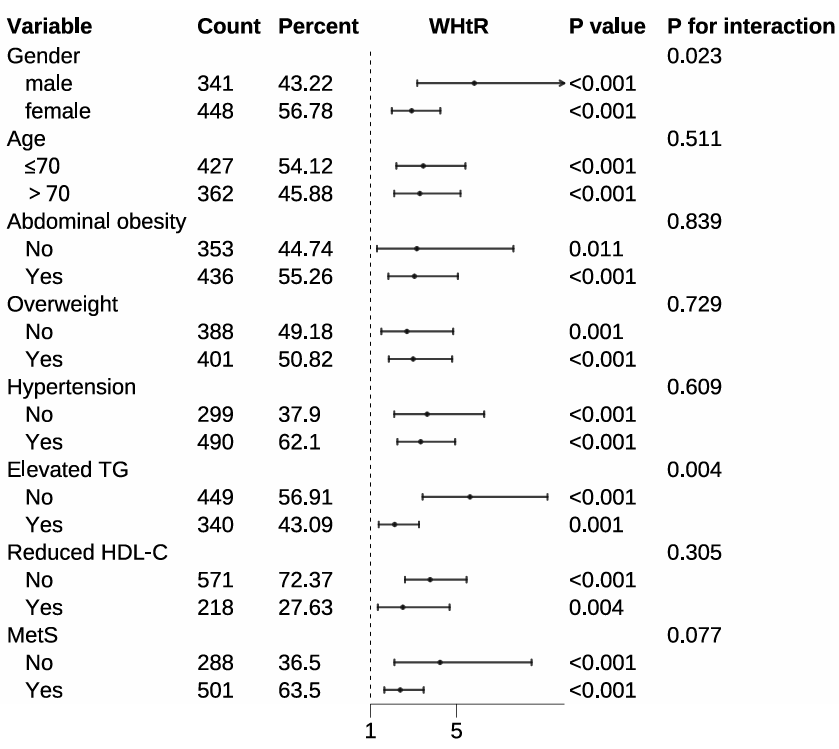


Figure S3 Subgroup analysis of the association between WHtR and NAFLD risk in cross-sectional studies

All models were adjusted for sex, age, exercise frequency, smoking status, antihypertensive medication, antidiabetic medication, lipid-lowering medication, and hypertension, except for the stratification variable.

BMI, body mass index; TG, triglycerides; HDL-C, high-density lipoprotein cholesterol; MetS, metabolic syndrome.


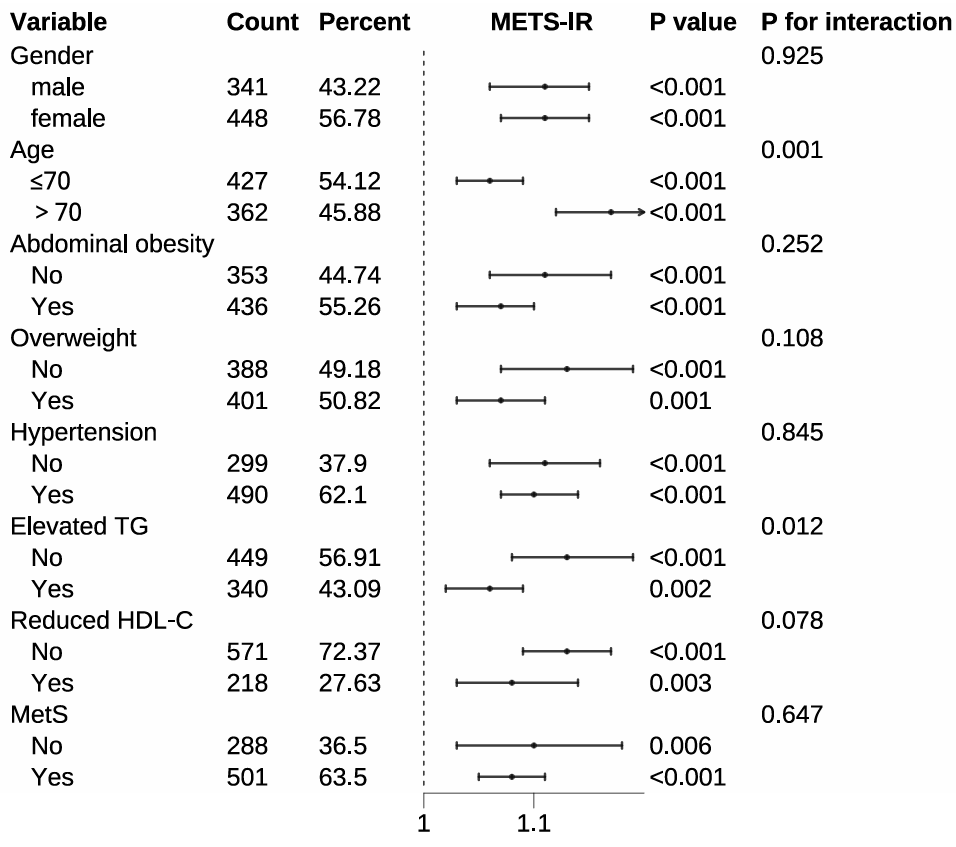


Figure S4 Subgroup analysis of the association between METS-IR and NAFLD risk in cross-sectional studies

All models were adjusted for sex, age, exercise frequency, smoking status, antihypertensive medication, antidiabetic medication, lipid-lowering medication, and hypertension, except for the stratification variable.

BMI, body mass index; TG, triglycerides; HDL-C, high-density lipoprotein cholesterol; MetS, metabolic syndrome.


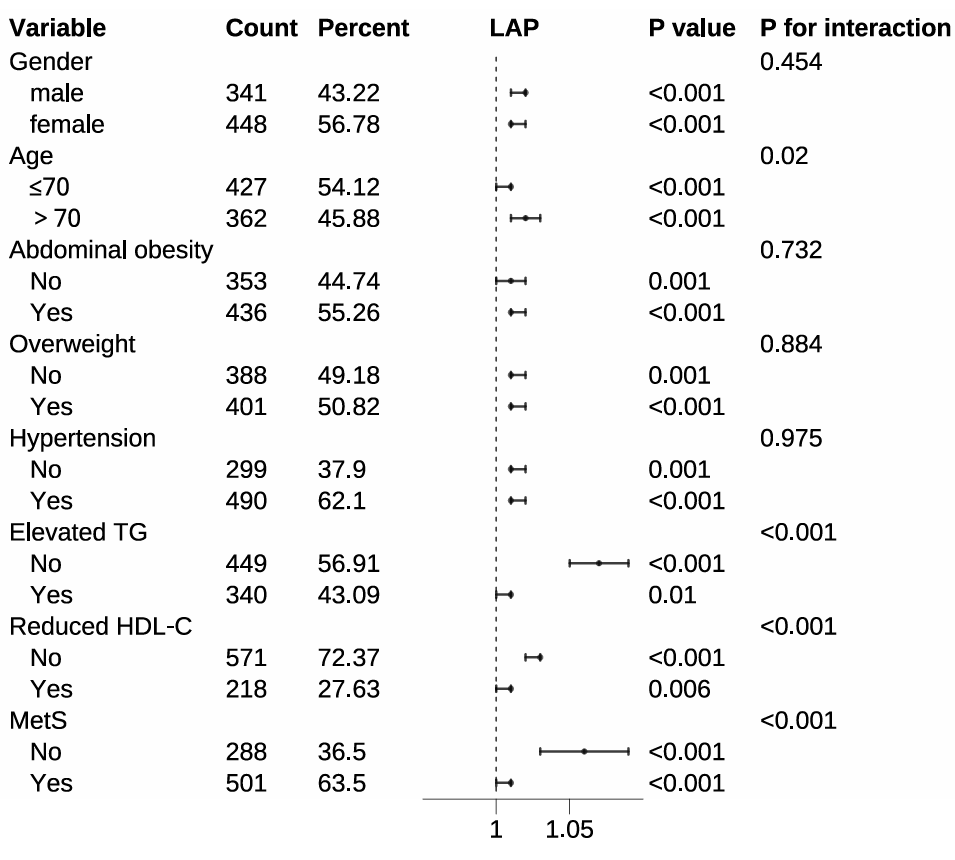


Figure S5 Subgroup analysis of the association between LAP and NAFLD risk in cross-sectional studies

All models were adjusted for sex, age, exercise frequency, smoking status, antihypertensive medication, antidiabetic medication, lipid-lowering medication, and hypertension, except for the stratification variable.

BMI, body mass index; TG, triglycerides; HDL-C, high-density lipoprotein cholesterol; MetS, metabolic syndrome.


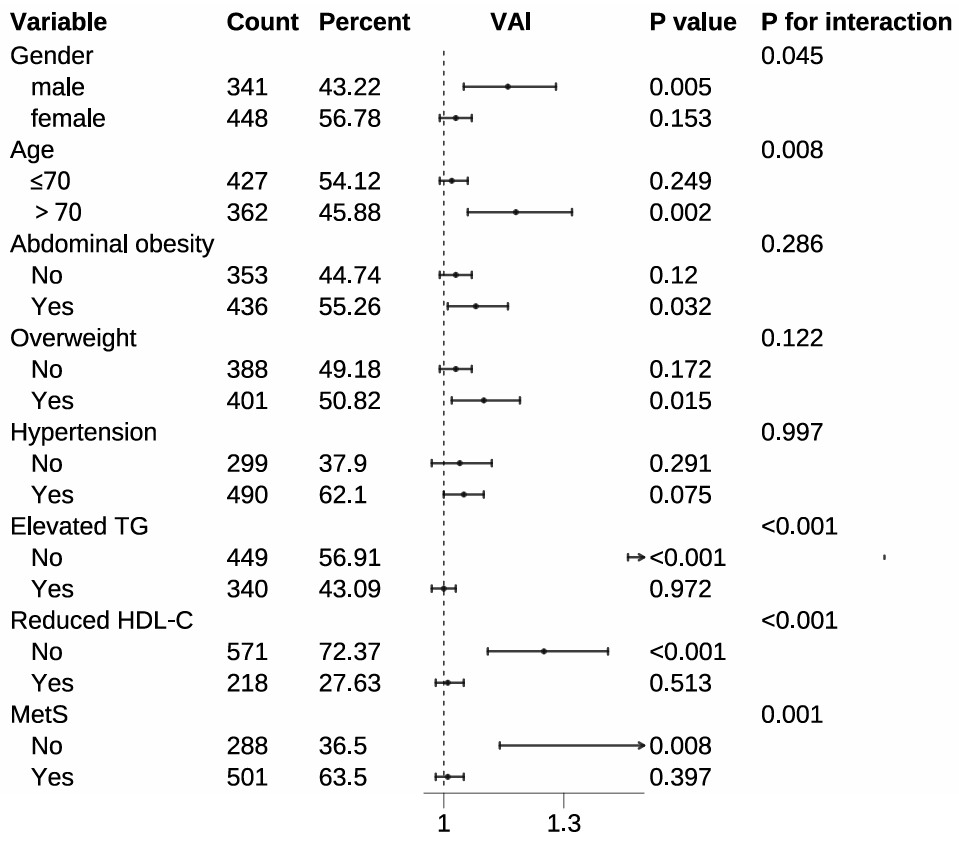


Figure S6 Subgroup analysis of the association between VAI and NAFLD risk in cross-sectional studies

All models were adjusted for sex, age, exercise frequency, smoking status, antihypertensive medication, antidiabetic medication, lipid-lowering medication, and hypertension, except for the stratification variable.

BMI, body mass index; TG, triglycerides; HDL-C, high-density lipoprotein cholesterol; MetS, metabolic syndrome.


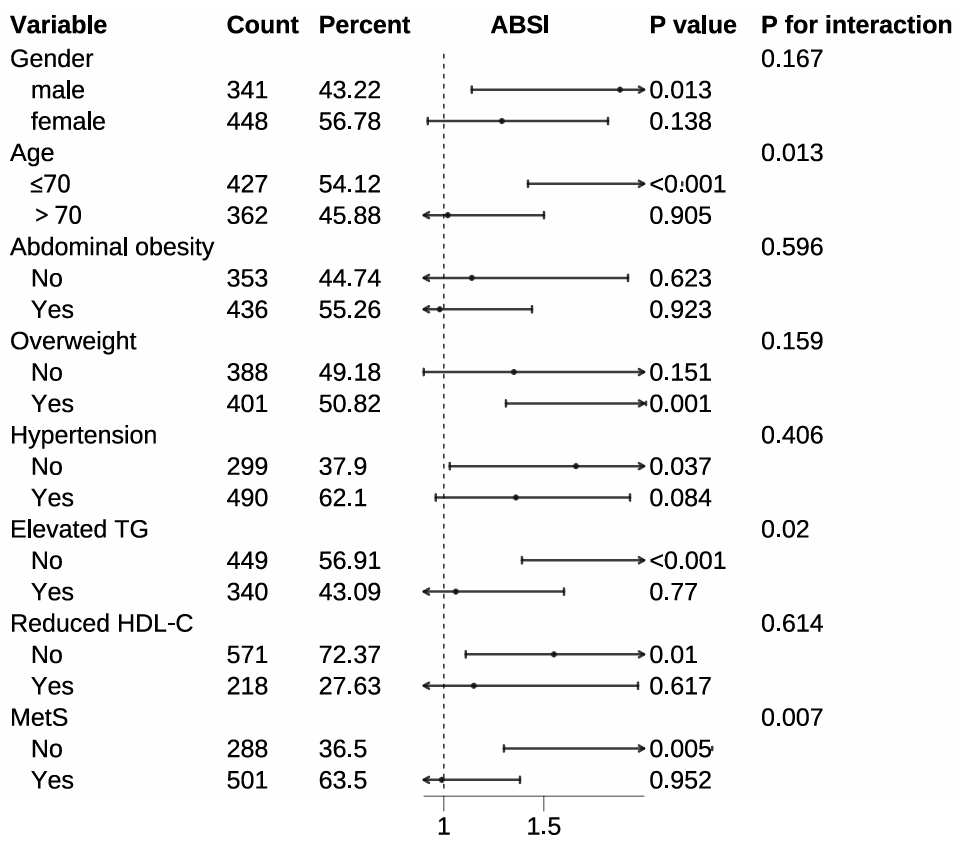


Figure S7 Subgroup analysis of the association between ABSI and NAFLD risk in cross-sectional studies

All models were adjusted for sex, age, exercise frequency, smoking status, antihypertensive medication, antidiabetic medication, lipid-lowering medication, and hypertension, except for the stratification variable.

BMI, body mass index; TG, triglycerides; HDL-C, high-density lipoprotein cholesterol; MetS, metabolic syndrome.


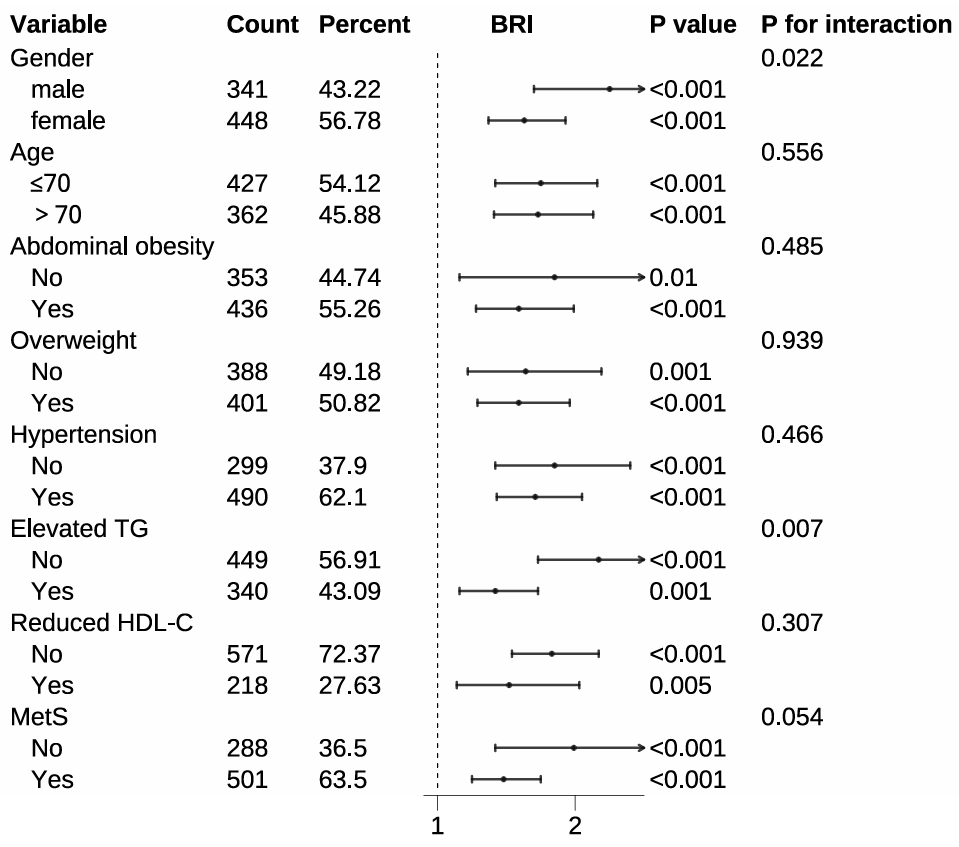


Figure S8 Subgroup analysis of the association between BRI and NAFLD risk in cross-sectional studies

All models were adjusted for sex, age, exercise frequency, smoking status, antihypertensive medication, antidiabetic medication, lipid-lowering medication, and hypertension, except for the stratification variable.

BMI, body mass index; TG, triglycerides; HDL-C, high-density lipoprotein cholesterol; MetS, metabolic syndrome.


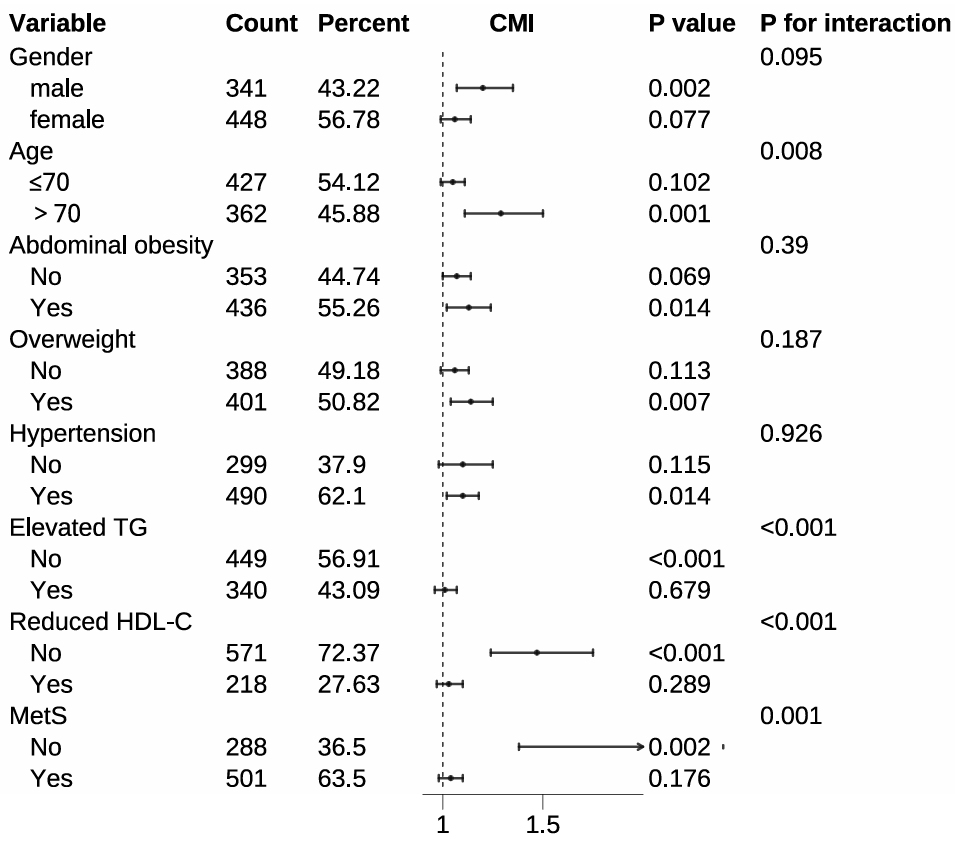


Figure S9 Subgroup analysis of the association between CMI and NAFLD risk in cross-sectional studies

All models were adjusted for sex, age, exercise frequency, smoking status, antihypertensive medication, antidiabetic medication, lipid-lowering medication, and hypertension, except for the stratification variable.

BMI, body mass index; TG, triglycerides; HDL-C, high-density lipoprotein cholesterol; MetS, metabolic syndrome.


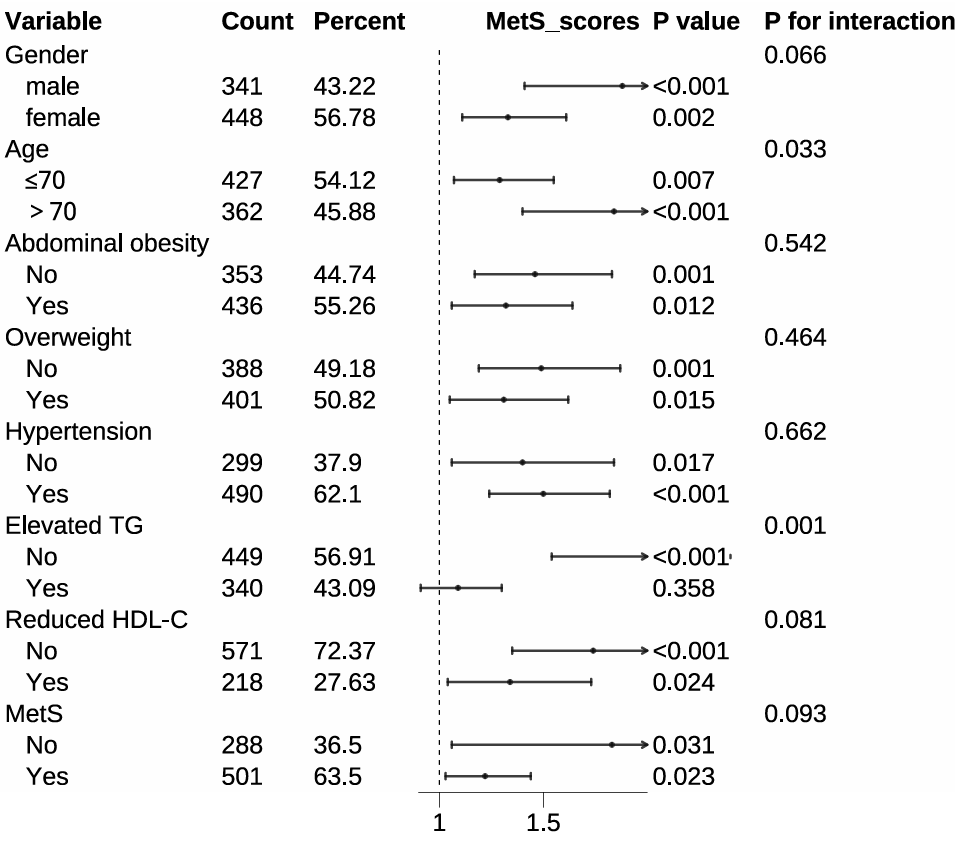


Figure S10 Subgroup analysis of the association between MetS scores and NAFLD risk in cross-sectional studies

All models were adjusted for sex, age, exercise frequency, smoking status, antihypertensive medication, antidiabetic medication, lipid-lowering medication, and hypertension, except for the stratification variable.

BMI, body mass index; TG, triglycerides; HDL-C, high-density lipoprotein cholesterol; MetS, metabolic syndrome.


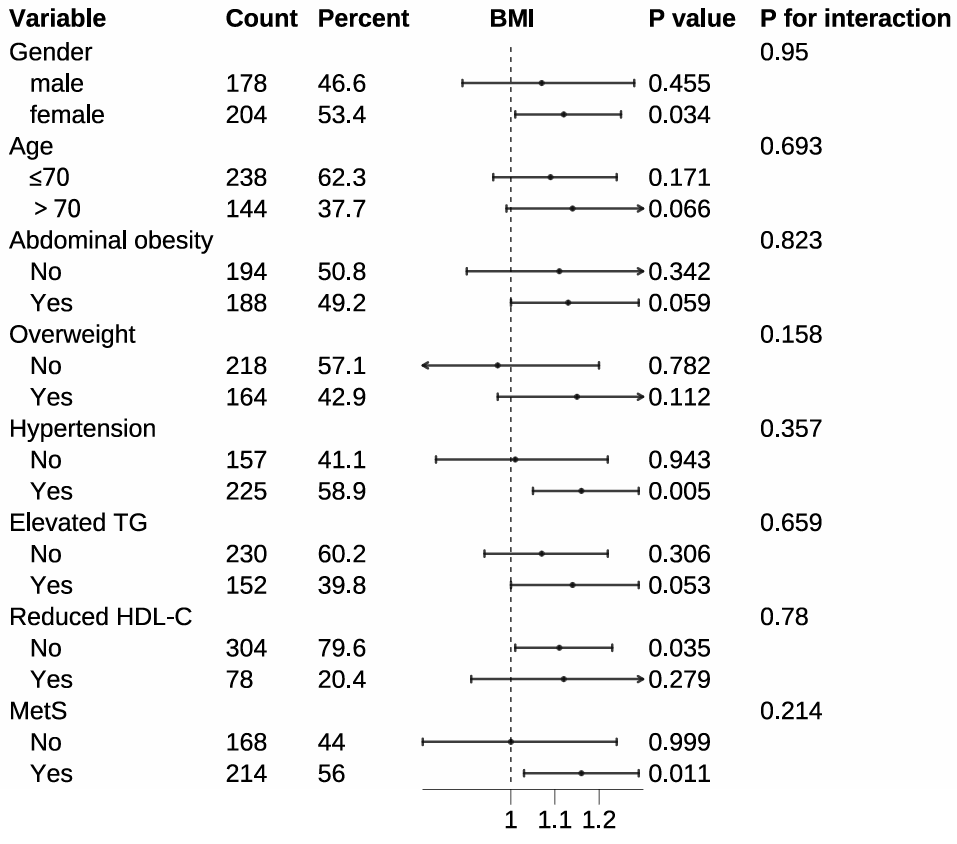


Figure S11 Subgroup analysis of the association between BMI and incident NAFLD in the longitudinal cohort

All models were adjusted for sex, age, exercise frequency, smoking status, antihypertensive medication, antidiabetic medication, lipid-lowering medication, and hypertension, except for the stratification variable.

BMI, body mass index; TG, triglycerides; HDL-C, high-density lipoprotein cholesterol; MetS, metabolic syndrome.


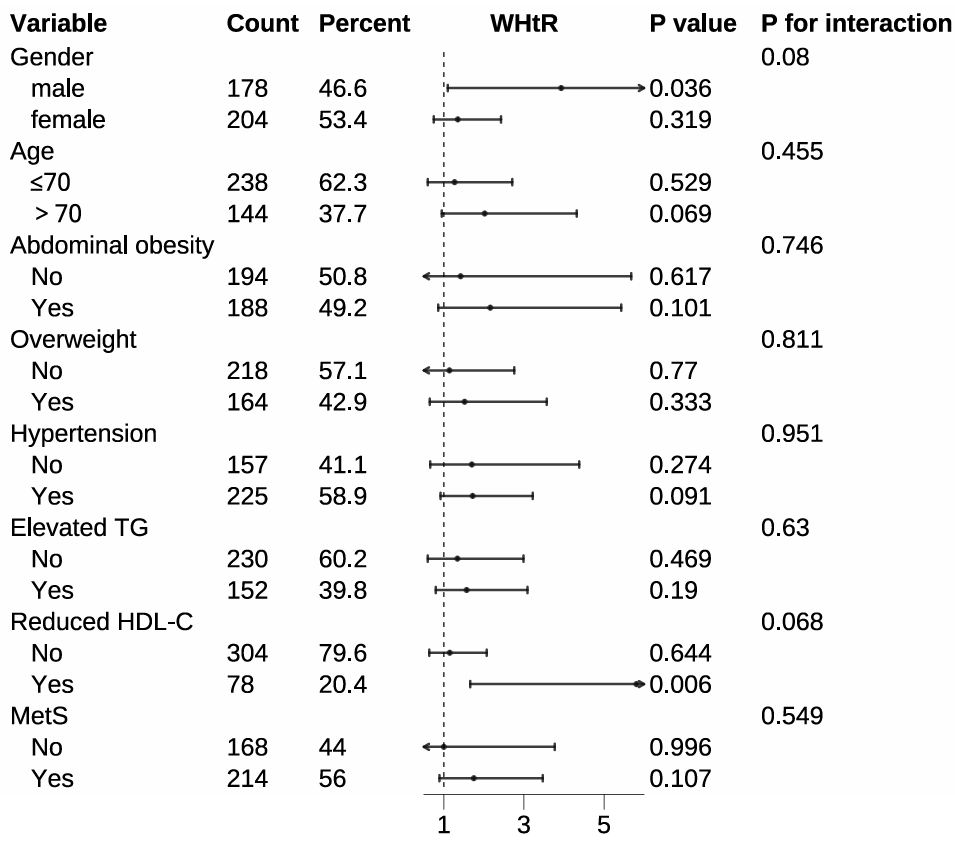


Figure S12 Subgroup analysis of the association between WHtR and incident NAFLD in the longitudinal cohort

All models were adjusted for sex, age, exercise frequency, smoking status, antihypertensive medication, antidiabetic medication, lipid-lowering medication, and hypertension, except for the stratification variable.

BMI, body mass index; TG, triglycerides; HDL-C, high-density lipoprotein cholesterol; MetS, metabolic syndrome.


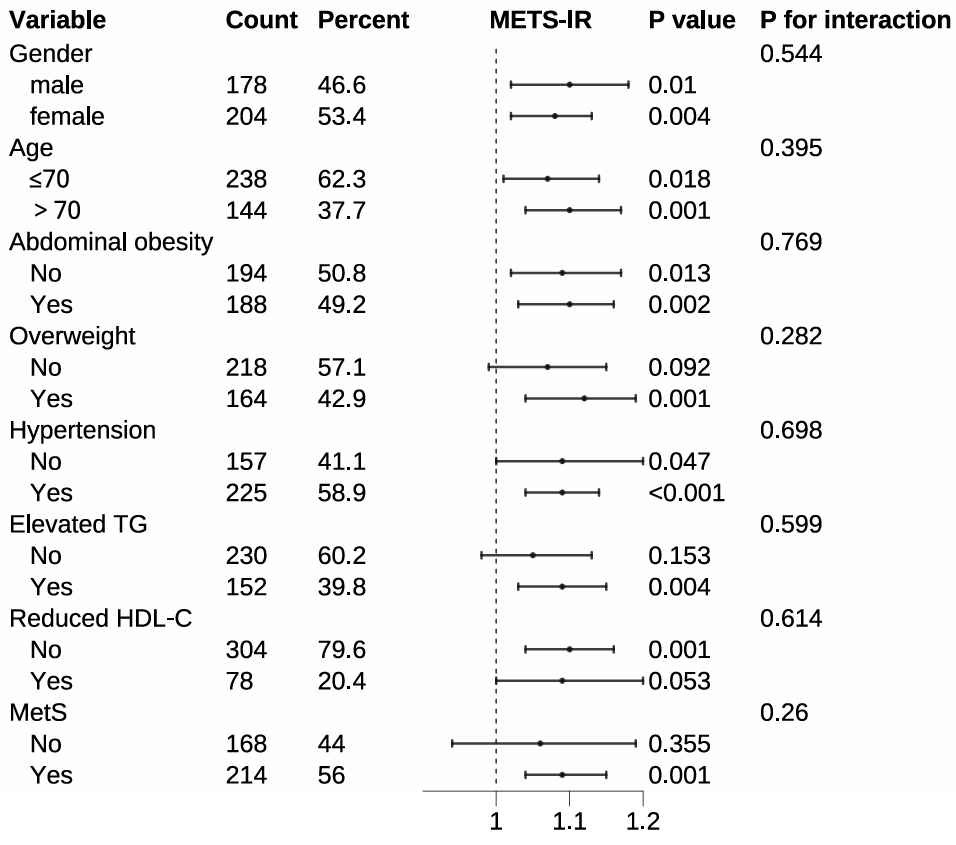


Figure S13 Subgroup analysis of the association between METS-IR and incident NAFLD in the longitudinal cohort

All models were adjusted for sex, age, exercise frequency, smoking status, antihypertensive medication, antidiabetic medication, lipid-lowering medication, and hypertension, except for the stratification variable.

BMI, body mass index; TG, triglycerides; HDL-C, high-density lipoprotein cholesterol; MetS, metabolic syndrome.


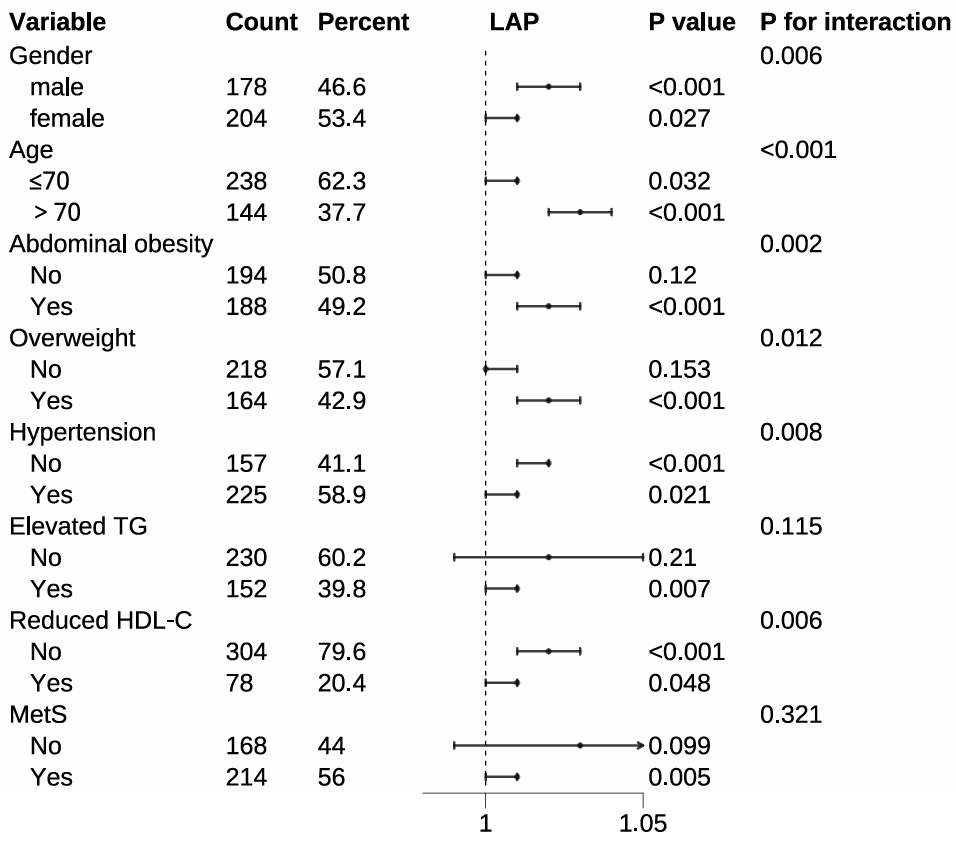


Figure S14 Subgroup analysis of the association between LAP and incident NAFLD in the longitudinal cohort

All models were adjusted for sex, age, exercise frequency, smoking status, antihypertensive medication, antidiabetic medication, lipid-lowering medication, and hypertension, except for the stratification variable.

BMI, body mass index; TG, triglycerides; HDL-C, high-density lipoprotein cholesterol; MetS, metabolic syndrome.


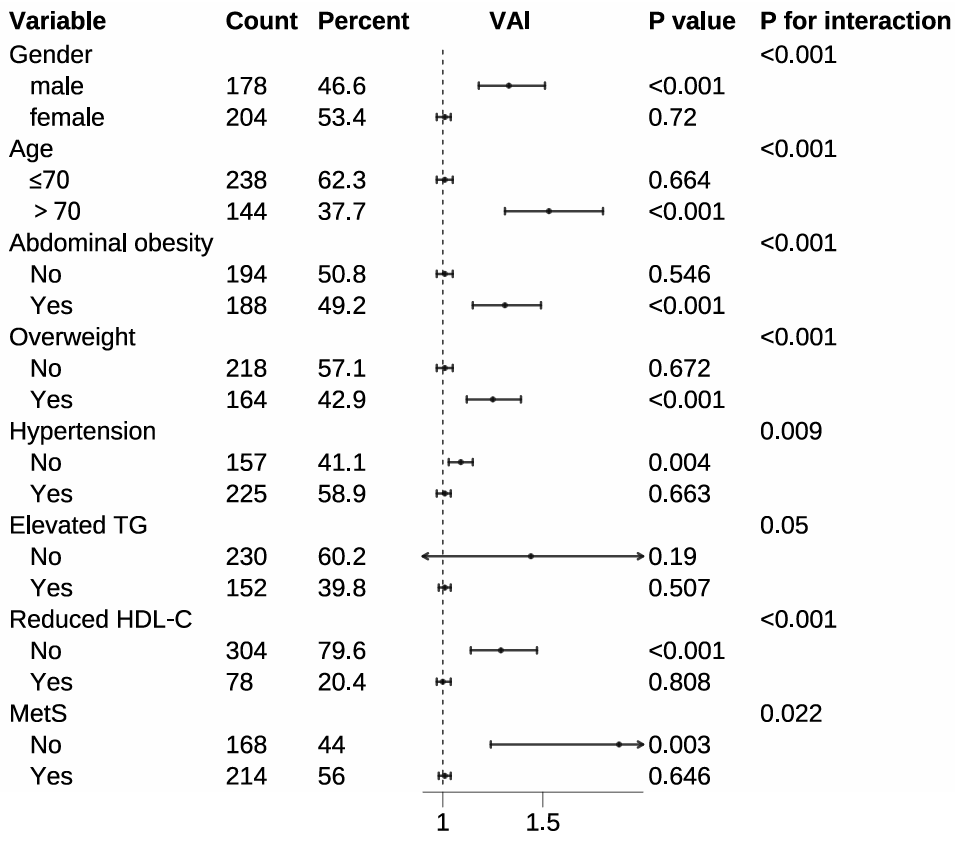


Figure S15 Subgroup analysis of the association between VAI and incident NAFLD in the longitudinal cohort

All models were adjusted for sex, age, exercise frequency, smoking status, antihypertensive medication, antidiabetic medication, lipid-lowering medication, and hypertension, except for the stratification variable.

BMI, body mass index; TG, triglycerides; HDL-C, high-density lipoprotein cholesterol; MetS, metabolic syndrome.


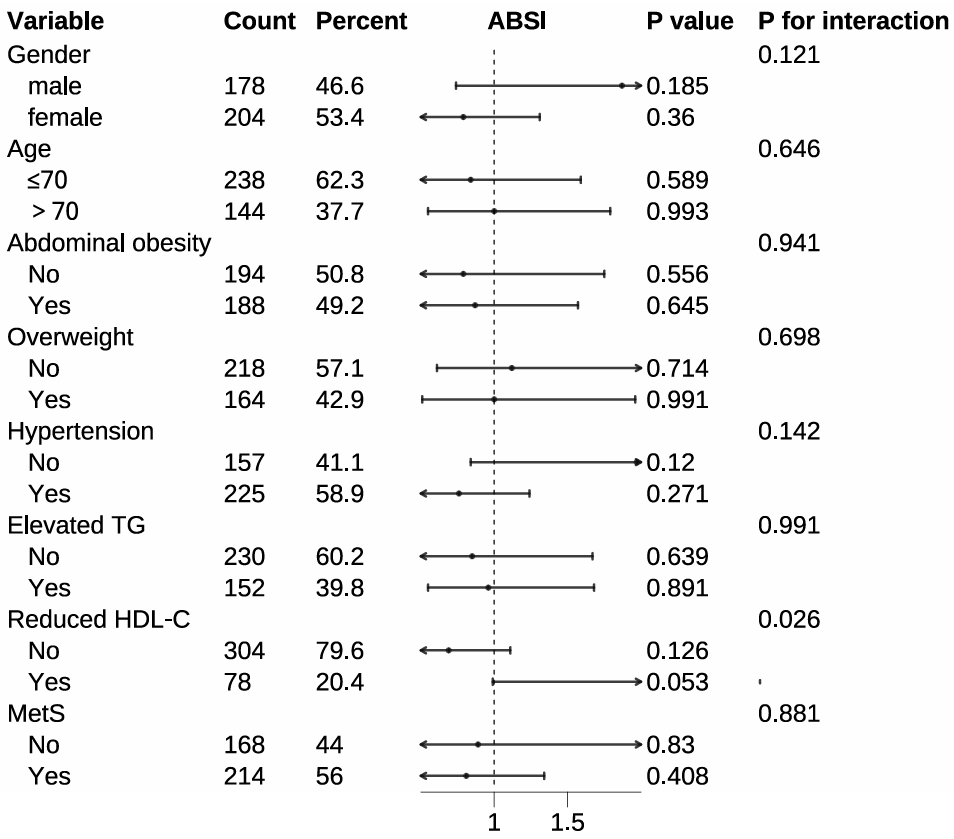


Figure S16 Subgroup analysis of the association between ABSI and incident NAFLD in the longitudinal cohort

All models were adjusted for sex, age, exercise frequency, smoking status, antihypertensive medication, antidiabetic medication, lipid-lowering medication, and hypertension, except for the stratification variable.

BMI, body mass index; TG, triglycerides; HDL-C, high-density lipoprotein cholesterol; MetS, metabolic syndrome.


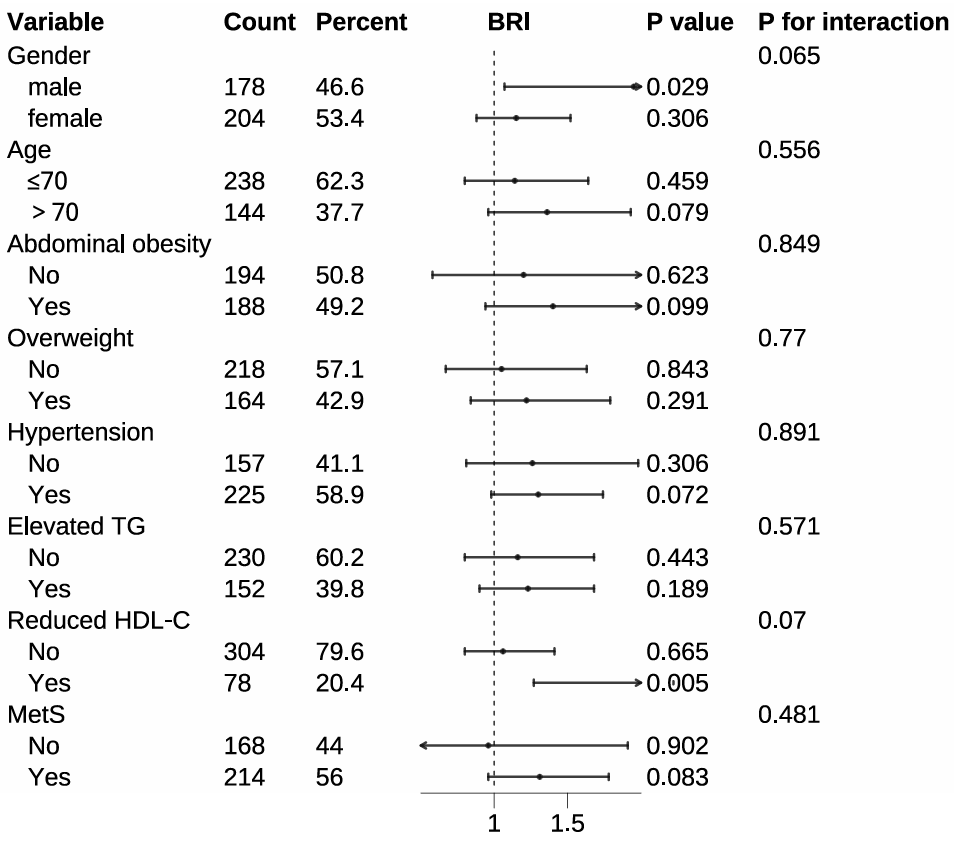


Figure S17 Subgroup analysis of the association between BRI and incident NAFLD in the longitudinal cohort

All models were adjusted for sex, age, exercise frequency, smoking status, antihypertensive medication, antidiabetic medication, lipid-lowering medication, and hypertension, except for the stratification variable.

BMI, body mass index; TG, triglycerides; HDL-C, high-density lipoprotein cholesterol; MetS, metabolic syndrome.


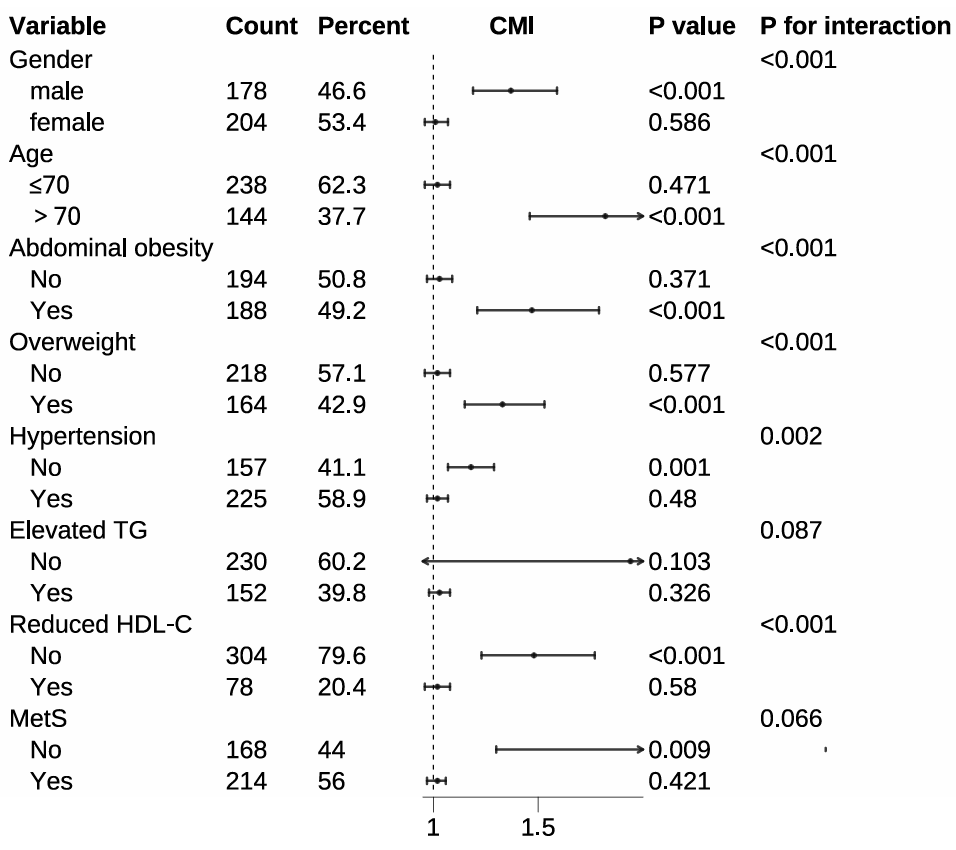


Figure S18 Subgroup analysis of the association between CMI and incident NAFLD in the longitudinal cohort

All models were adjusted for sex, age, exercise frequency, smoking status, antihypertensive medication, antidiabetic medication, lipid-lowering medication, and hypertension, except for the stratification variable.

BMI, body mass index; TG, triglycerides; HDL-C, high-density lipoprotein cholesterol; MetS, metabolic syndrome.


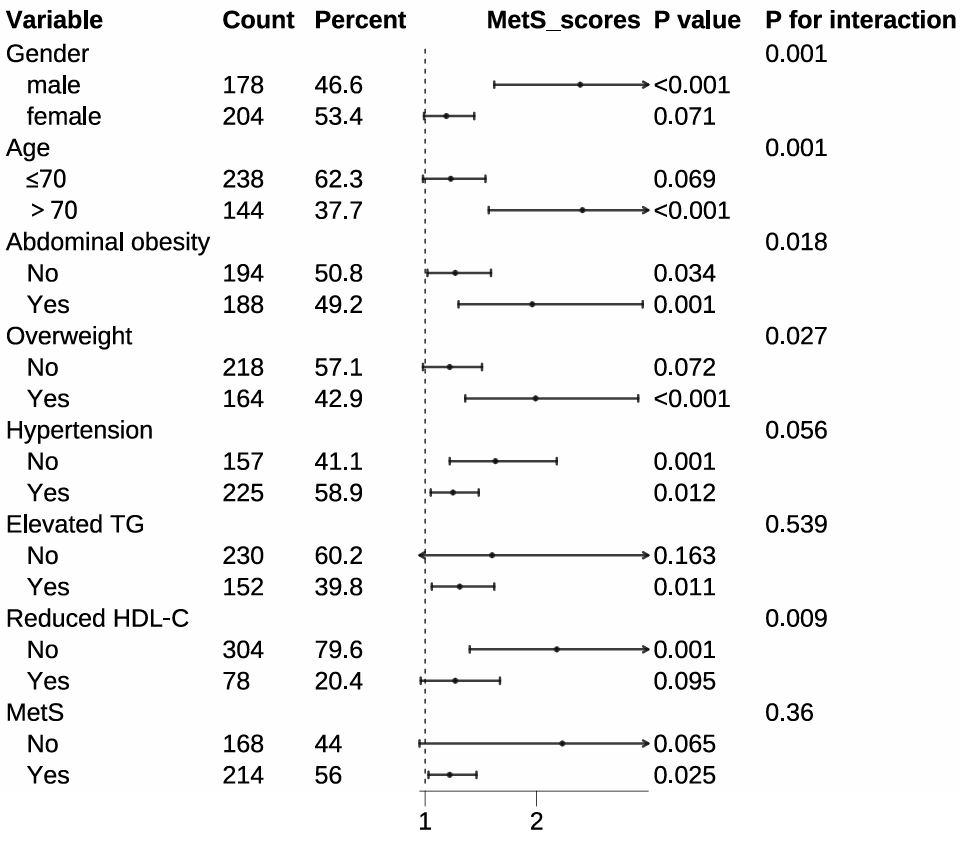


Figure S19 Subgroup analysis of the association between MetS scores and incident NAFLD in the longitudinal cohort

All models were adjusted for sex, age, exercise frequency, smoking status, antihypertensive medication, antidiabetic medication, lipid-lowering medication, and hypertension, except for the stratification variable.

BMI, body mass index; TG, triglycerides; HDL-C, high-density lipoprotein cholesterol; MetS, metabolic syndrome.


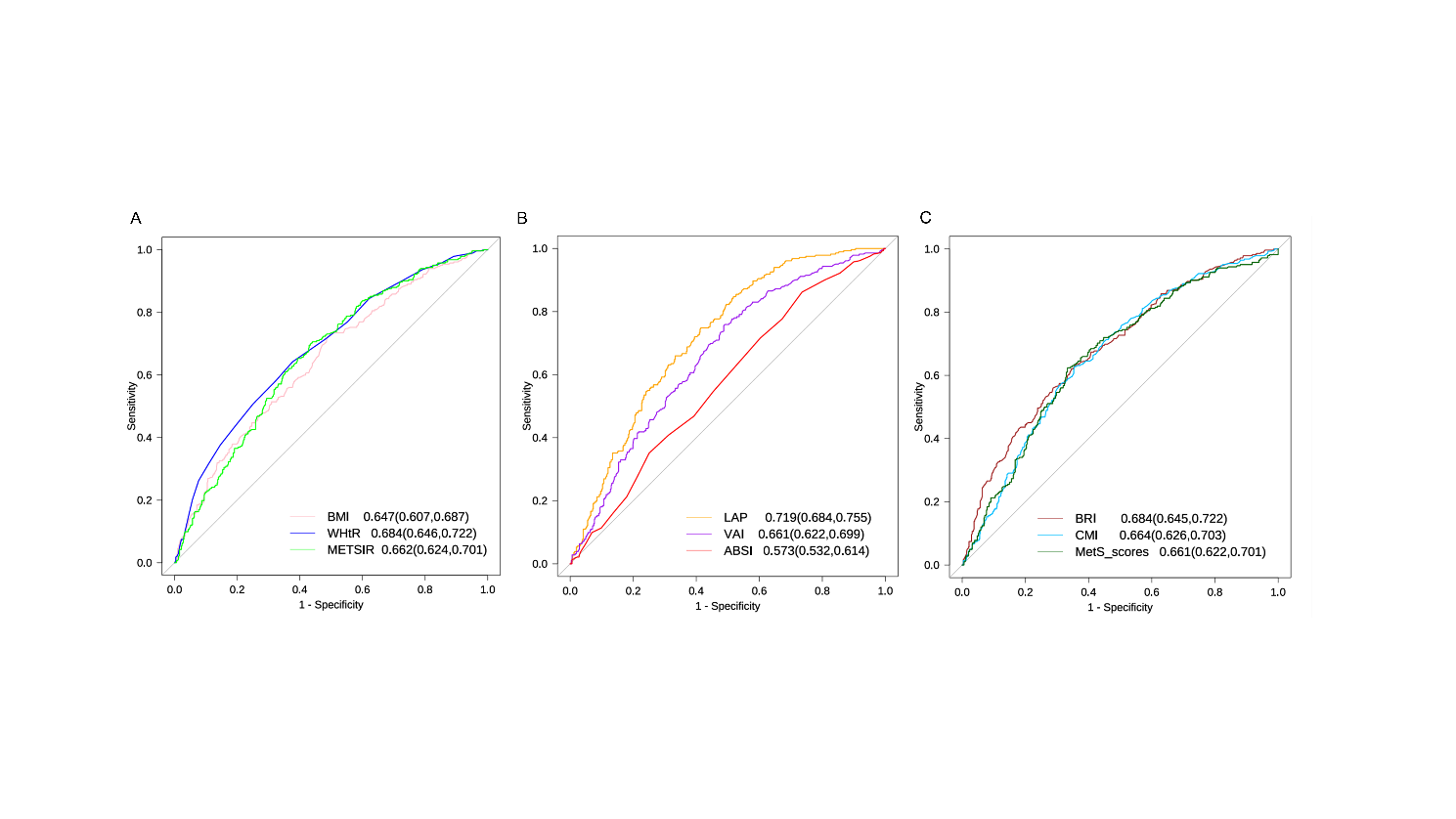


Figure S20 Discriminative performance of metabolic indices for NAFLD in cross-sectional analysis


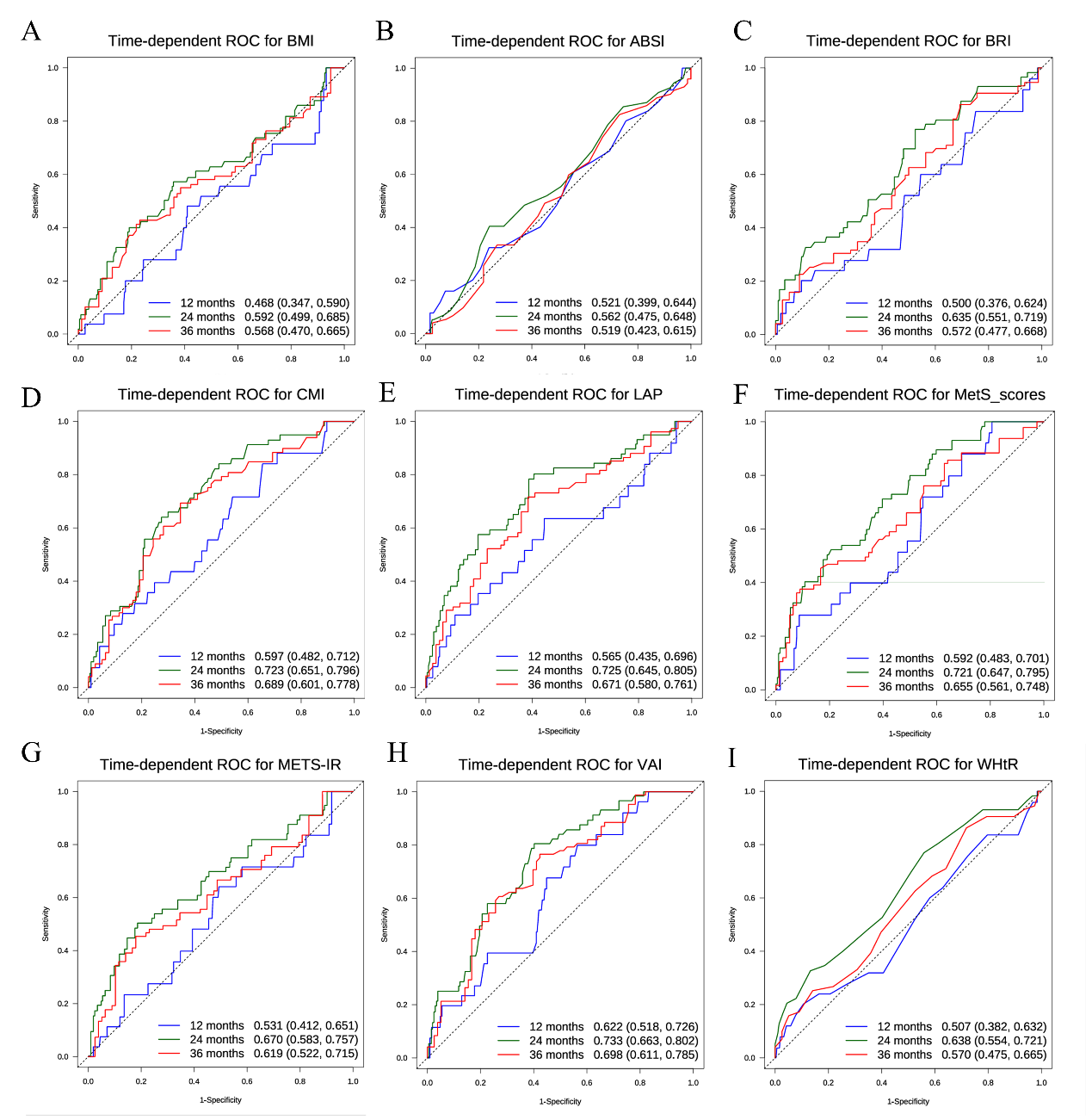


Figure S21 Time-dependent ROC curves of metabolic indices for predicting incident NAFLD at 12, 24, and 36 months


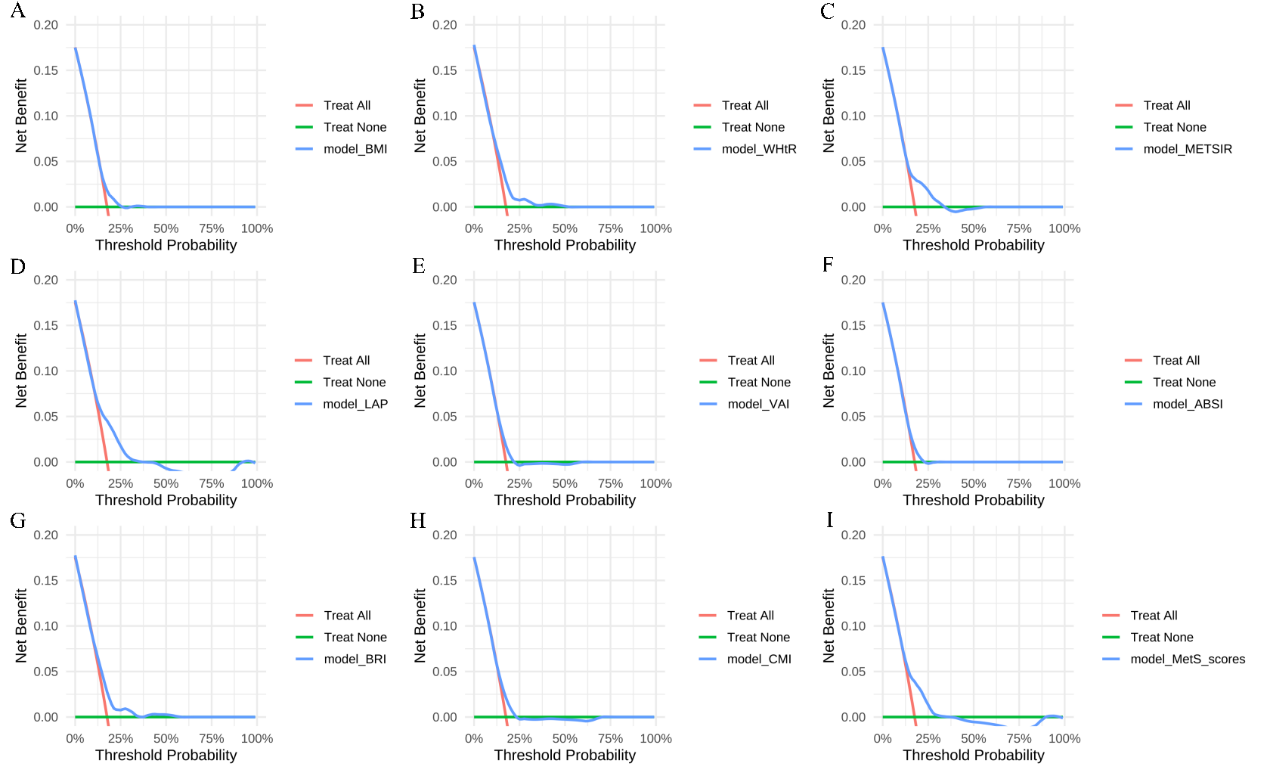


Figure S22 Decision Curve Analysis of Obesity- and Metabolism-Related Predictors for NAFLD
